# SARS-CoV-2 Causes Severe Epithelial Inflammation and Barrier Dysfunction

**DOI:** 10.1128/JVI.00110-21

**Published:** 2021-04-26

**Authors:** Stefanie Deinhardt-Emmer, Sarah Böttcher, Clio Häring, Liane Giebeler, Andreas Henke, Roland Zell, Johannes Jungwirth, Paul M. Jordan, Oliver Werz, Franziska Hornung, Christian Brandt, Mike Marquet, Alexander S. Mosig, Mathias W. Pletz, Michael Schacke, Jürgen Rödel, Regine Heller, Sandor Nietzsche, Bettina Löffler, Christina Ehrhardt

**Affiliations:** a Institute of Medical Microbiology, Jena University Hospital, Jena, Germany; b Section of Experimental Virology, Institute of Medical Microbiology, Jena University Hospital, Jena, Germany; c Center for Sepsis Control and Care, Jena University Hospital, Jena, Germany; d Department of Pharmaceutical/Medicinal Chemistry, Institute of Pharmacy, Friedrich-Schiller-University Jena, Jena, Germany; e Institute for Infectious Diseases and Infection Control, Jena University Hospital, Jena, Germany; f Institute of Biochemistry, Jena University Hospital, Jena, Germany; g Institute of Molecular Cell Biology, Center for Molecular Biomedicine (CMB), Jena University Hospital, Jena, Germany; h Center for Electron Microscopy, Jena University Hospital, Jena, Germany; Loyola University Chicago

**Keywords:** COVID-19, SARS-CoV-2, chip model, epithelial/endothelial barrier, interferons

## Abstract

Infections with severe acute respiratory syndrome coronavirus 2 (SARS-CoV-2) can be asymptomatic, but they can also be accompanied by a variety of symptoms that result in mild to severe coronavirus disease-19 (COVID-19) and are sometimes associated with systemic symptoms. Although the viral infection originates in the respiratory system, it is unclear how the virus can overcome the alveolar barrier, which is observed in severe COVID-19 disease courses. To elucidate the viral effects on the barrier integrity and immune reactions, we used mono-cell culture systems and a complex human chip model composed of epithelial, endothelial, and mononuclear cells. Our data show that SARS-CoV-2 efficiently infected epithelial cells with high viral loads and inflammatory response, including interferon expression. In contrast, the adjacent endothelial layer was neither infected nor did it show productive virus replication or interferon release. With prolonged infection, both cell types were damaged, and the barrier function deteriorated, allowing the viral particles to overbear. In our study, we demonstrate that although SARS-CoV-2 is dependent on the epithelium for efficient replication, the neighboring endothelial cells are affected, e.g., by the epithelial cytokines or components induced during infection, which further results in damage of the epithelial/endothelial barrier function and viral dissemination.

**IMPORTANCE** SARS-CoV-2 challenges health care systems and societies worldwide in unprecedented ways. Although numerous new studies have been conducted, research to better understand the molecular pathogen-host interactions is urgently needed. For this, experimental models have to be developed and adapted. In the present study we used mono-cell culture systems, and we established a complex chip model, where epithelial and endothelial cells are cultured in close proximity. We demonstrate that epithelial cells can be infected with SARS-CoV-2, while the endothelium did not show any infection signs. Since SARS-CoV-2 is able to establish viremia, the link to thromboembolic events in severe COVID-19 courses is evident. However, whether the endothelial layer is damaged by the viral pathogens or whether other endothelial-independent homeostatic factors are induced by the virus is essential for understanding the disease development. Therefore, our study is important, as it demonstrates that the endothelial layer could not be infected by SARS-CoV-2 in our *in vitro* experiments, but we were able to show the destruction of the epithelial-endothelial barrier in our chip model. From our experiments, we can assume that virus-induced host factors disturbed the epithelial-endothelial barrier function and thereby promote viral spread.

## Abstract (precis)

SARS-CoV-2 challenges health care systems and societies worldwide in unprecedented ways. Although numerous new studies have been conducted, research to better understand the molecular pathogen-host interactions are urgently needed.

## INTRODUCTION

The novel severe acute respiratory syndrome coronavirus 2 (SARS-CoV-2) is a highly pathogenic virus causing severe respiratory infections, described as coronavirus disease-19 (COVID-19) (1). Patients suffer from various symptoms such as fever, cough, shortness of breath, headache, muscle aches, and gastrointestinal symptoms. Hallmarks of severe COVID-19 courses are pneumonia, pulmonary edema, acute respiratory distress syndrome (ARDS), and multiple organ failure. In most patients, the disease has a mild course, but in some cases, e.g., elderly with comorbidities, the infection can develop into a life-threatening condition. In particular, preexisting lung pathologies and systemic diseases such as diabetes predispose to severe infection courses described by George et al. (2).

Clinical studies revealed that the virus primarily replicates in the lung, which can cause severe lung damage up to necrotic destruction of large areas of the lung tissue (3). In autopsies of deceased COVID-19 patients, it has been observed that particularly in severe cases, viral particles can disseminate throughout the body (4, 5). Additionally, systemic complications have been reported, such as the massive release of proinflammatory cytokines and thromboembolic events in various organs (6). Consequently, SARS-CoV-2 is regarded as a virus that primarily replicates in the nasopharyngeal area but can also cause a systemic infection that affects different organs with a high mortality rate.

Little is known about the initial infection process in the alveolar lung tissue, particularly about mechanisms that destroy the lung and mechanisms that allow the virus to affect different organs in the body. *In vivo* infection models that closely reflect the patient’s situation are largely lacking, in part due to the different disease development in mice and humans. Furthermore, extensive safety measures are required to infect mice with SARS-CoV-2. Up to now, SARS-CoV-2 *in vitro* infection models have been mainly performed with human airway (nonalveolar) cells or nonhuman cell lines that naturally express the ACE2 viral receptor, such as the African green monkey Vero-76 cell line (7). These cells lack organ- and species-specific characteristics of human lung epithelial cells. For this purpose, cancerous human lung epithelial cells (Calu-3 cells) can, at least to some extent, reflect the response of the lung epithelium to viral infection (8).

In our study, we present a human-specific *in vitro* chip model composed of cells of human origin susceptible to a SARS-CoV-2 infection. This model was only recently developed in our lab (9). In the present study, it was modified by using SARS-CoV-2 permissive epithelial cells (Calu-3 cells). The epithelial and vascular cells (primarily isolated human umbilical vascular endothelial cells; HUVECs) were cocultured with macrophages (primarily isolated peripheral blood mononuclear cells; PBMCs), building a complex cell culture system. This composition is relevant not only for the gas exchange in the lung but also for an adequate immune response.

We were able to show that SARS-CoV-2 replicates in the epithelial layer while inducing an acute and robust inflammatory response followed by the destruction of the epithelial layer. Interestingly, in this infection scenario, the endothelial cells were not invaded by SARS-CoV-2 and did not propagate the virus, but nevertheless, the epithelial/endothelial barrier integrity was disrupted.

## RESULTS

### Efficient SARS-CoV-2 isolation from patients and propagation in cell culture.

To gain fully infectious viral particles for our studies, we collected three respiratory specimens from reverse transcription-quantitative PCR (qRT-PCR)-proven COVID-19 patients and performed SARS-CoV-2 propagation in cell culture systems (Vero-76 cells). By repeated infection of host cells and viral replication, we were able to isolate high viral titers originating from three different patients. Within our studies, the SARS-CoV-2 isolates SARS-CoV-2/hu/Germany/Jena-vi005159/2020 (5159), SARS-CoV-2/hu/Germany/Jena-vi005587/2020 (5587), and SARS-CoV-2/hu/Germany/Jena-vi005588/2020 (5588) were employed. Sequencing of virus isolates verified that all three viral strains belong to SARS-CoV-2 (species *Severe acute respiratory syndrome-related coronavirus*, genus *Betacoronavirus*) (10). Phylogenetic analysis revealed a close relationship of SARS-CoV-2 to the SARS-related coronaviruses RaTG13, bat-SL-CoVZXC21, and bat-SL-CoVZC45 (see Fig. S1A in the supplemental material). Within the SARS-CoV-2 clade, the sequences of strains 5587 and 5588 exhibit two base substitutions T8,782C (nsp1ab: synonymous) and C28,144T (nsp8: S84L), which are characteristic for all strains of lineage L (nomenclature [11]) or lineage B (nomenclature [12]). Accordingly, 5587 and 5588 clustered with lineage L/lineage B strains in the phylogenetic analysis (Fig. S1B). Furthermore, both strains exhibit deletion of nsp1ab D448 and two synonymous substitutions (T514C, C5512T). Beside the nsp8 S84L substitution, strain 5159 has accumulated three additional amino acid substitutions (S, D614G; nsp1ab, P4715L; and N, R203K/G204R), which place this virus in lineage B.1.1 according to the proposed SARS-CoV-2 nomenclature of Rambaut et al. (12) (Fig. S1B).

### Mono-cell culture systems can be infected by SARS-CoV-2 and produce replication complexes at ER-derived membranes.

**FIG 1:**
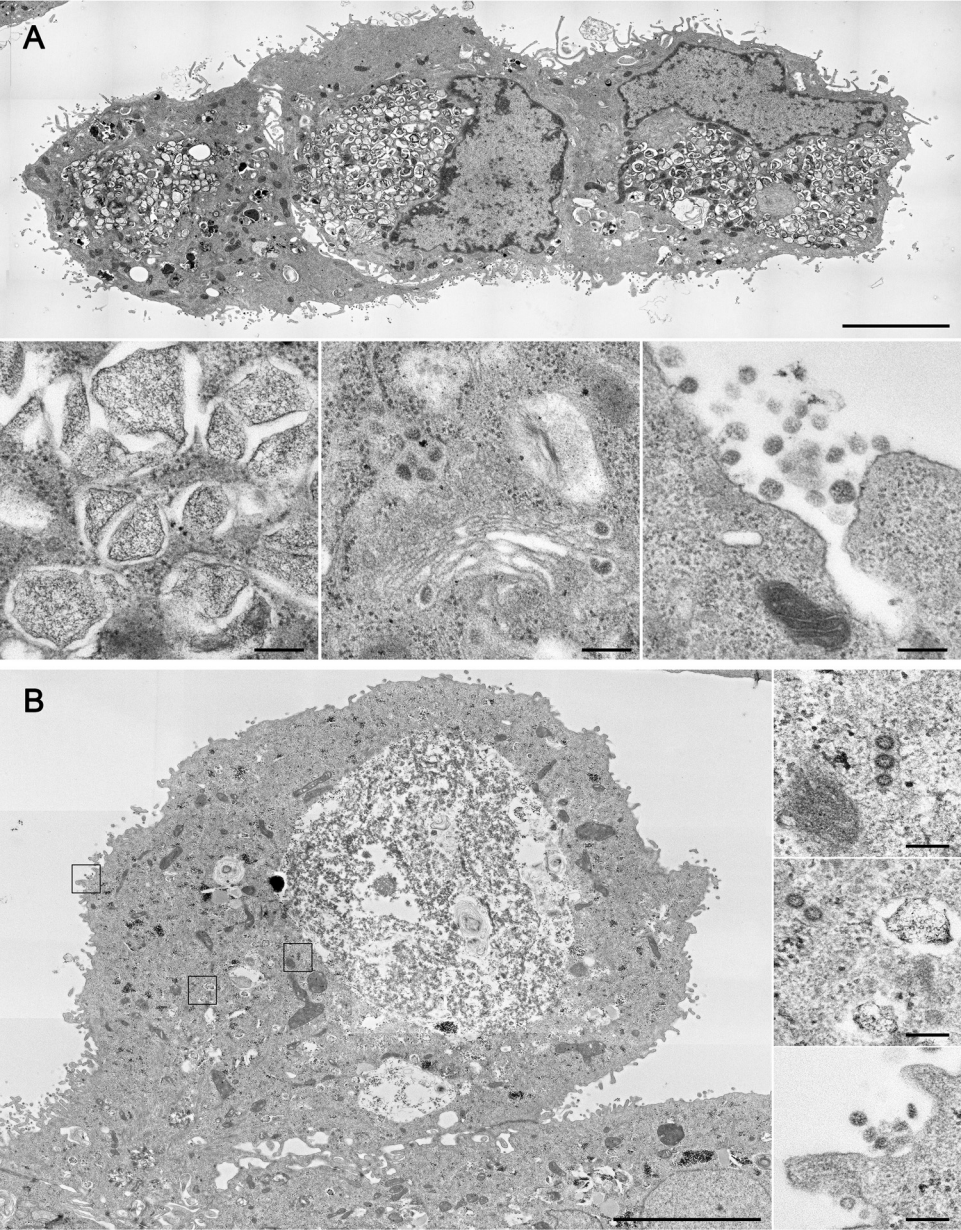
Transmission electron microscopy of SARS-CoV-2-infected Vero-76 and Calu-3 cells. Vero-76 (A) and Calu-3 (B) cells were infected with a SARS-CoV-2 patient isolate (5159, MOI = 1). Transmission electron microscopy was performed 24 h postinfection (p.i.). (A) (Top panel; scale bar, 5 μm) overview of 3 SARS-CoV-2-infected Vero-76 cells; (bottom-left panel; scale bar, 200 nm) generation of double membrane vesicles; (bottom-middle panel; scale bar, 200 nm) virion assembly in the ER–Golgi-intermediate compartment (ERGIC); (bottom-right panel; scale bar: 200 nm) viral release. (B) Overview of a SARS-CoV-2-infected Calu-3 cell showing virus replication activity (large panel; scale bar, 5 μm); in the close-ups, transportation of assembled virions (top-right panel; scale bar, 200 nm), double membrane vesicles (middle-right panel; scale bar, 200 nm), and virus particle release (bottom-right panel; scale bar, 200 nm) are shown.

First, we infected mono-cell culture systems with SARS-CoV-2 and compared the infection rate between Vero-76 cells and Calu-3 cells. It is already well known that Vero-76 cells can be efficiently infected by SARS-CoV-2 (7, 13). To better mimic the situation in the human lung, we performed the infection in Calu-3 cells. Using transmission electron microscopy (TEM), we demonstrated that Vero-76 and Calu-3 cells efficiently propagate the virus (Fig. 1). Figure 1 illustrates infected Vero-76 (Fig. 1A) and Calu-3 cells (Fig. 1B) containing viral replication organelles. In the closeup panels, protein accumulation and generation of double-membrane vesicles are visible in both cell types. In the middle panel (Fig. 1A), virion assembly in the endoplasmic reticulum-Golgi intermediate compartment (ERGIC) and a Golgi complex are imaged containing morphologically complete viral particles. Here, the particles are packed to be transported to the cellular surface for virus release. In both cell types, we see that some particles are still attached to the host cell membrane, whereas some viral particles are already fully released (lower right panels). These results indicate that SARS-CoV-2 induces replication complexes at ER-derived membranes, which were already shown for other types of coronaviruses (14) and which confirms the findings for Vero E6 cells (15).

**FIG 2:**
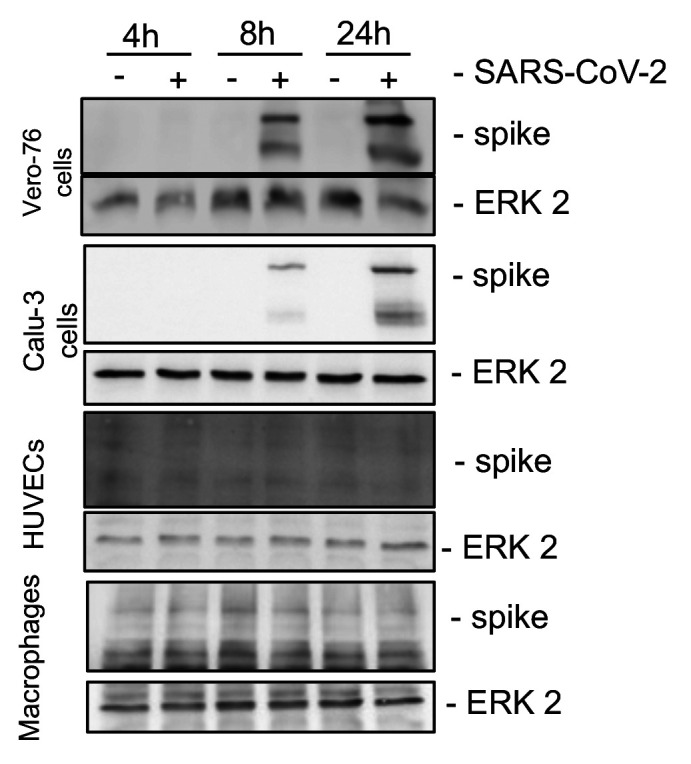
SARS-CoV-2 replicates in Vero-76 and Calu-3 cells. Vero-76, Calu-3 cells, HUVECs, and macrophages were left uninfected (mock) (A, B) or were infected (A-D) with a SARS-CoV-2 patient isolate (5159) (A, B) or (5159, 5587, 5588) (C, D) (MOI, 1). (A) SARS-CoV-2 was visualized by detection of the spike protein via a spike-specific antibody and an Alexa Fluor 488-conjugated goat anti-mouse IgG (green). The nuclei were stained with Hoechst 33342 (blue). Immunofluorescence (IF) microscopy was acquired by use of the Axio Observer.Z1 instrument (Zeiss) with a ×200 magnification. (B) Total cell lysates were harvested at the times indicated, and expression of the spike protein was analyzed by Western blot analysis. ERK2 served as the loading control. (C) Progeny virus particles were measured in the supernatant with a standard plaque assay at the indicated times postinfection (p.i.). Shown are the means (±SD) of PFU ml^−1^ of at least three independent experiments, including two biological samples. Statistical significance was analyzed with unpaired, two-tailed *t* tests (***, *P* < 0.001). (D) RNA-lysates were performed 24 h p.i., and copies of viral RNA (E-gene) were determined with r-biopharm qRT-PCR. Means ± SD of three independent experiments are shown.

In Fig. 2A, immunofluorescence measurements compare infected Vero-76 cells with infected Calu-3 cells, HUVECs, and macrophages. Most remarkably, in Vero-76 and Calu-3 cells, viral particles can be visualized to a similar extent using specific antibodies against SARS-CoV-2 spike proteins. These results are confirmed by Western blot analysis, demonstrating increased levels of SARS-CoV-2 spike protein in the cell lysates after 8 h and 24 h, which is missing in HUVECs and macrophages (Fig. 2B).

Additionally, we verified progeny virus particles by performing plaque assays from supernatants taken at 8 h and 24 h postinfection. Our results demonstrate increased replication during ongoing infection of Vero-76 and Calu-3 cells, but no viral propagation was found in HUVECs and macrophages (Fig. 2C). Measuring viral RNA loads in different infected host cell types 24 h postinfection, we found high RNA levels only in Vero-76 and Calu-3 cells (Fig. 2D). Thus, HUVEC mono-cell cultures and macrophages could not productively be infected by SARS-CoV-2 (Fig. 2A to D).

**FIG 3:**
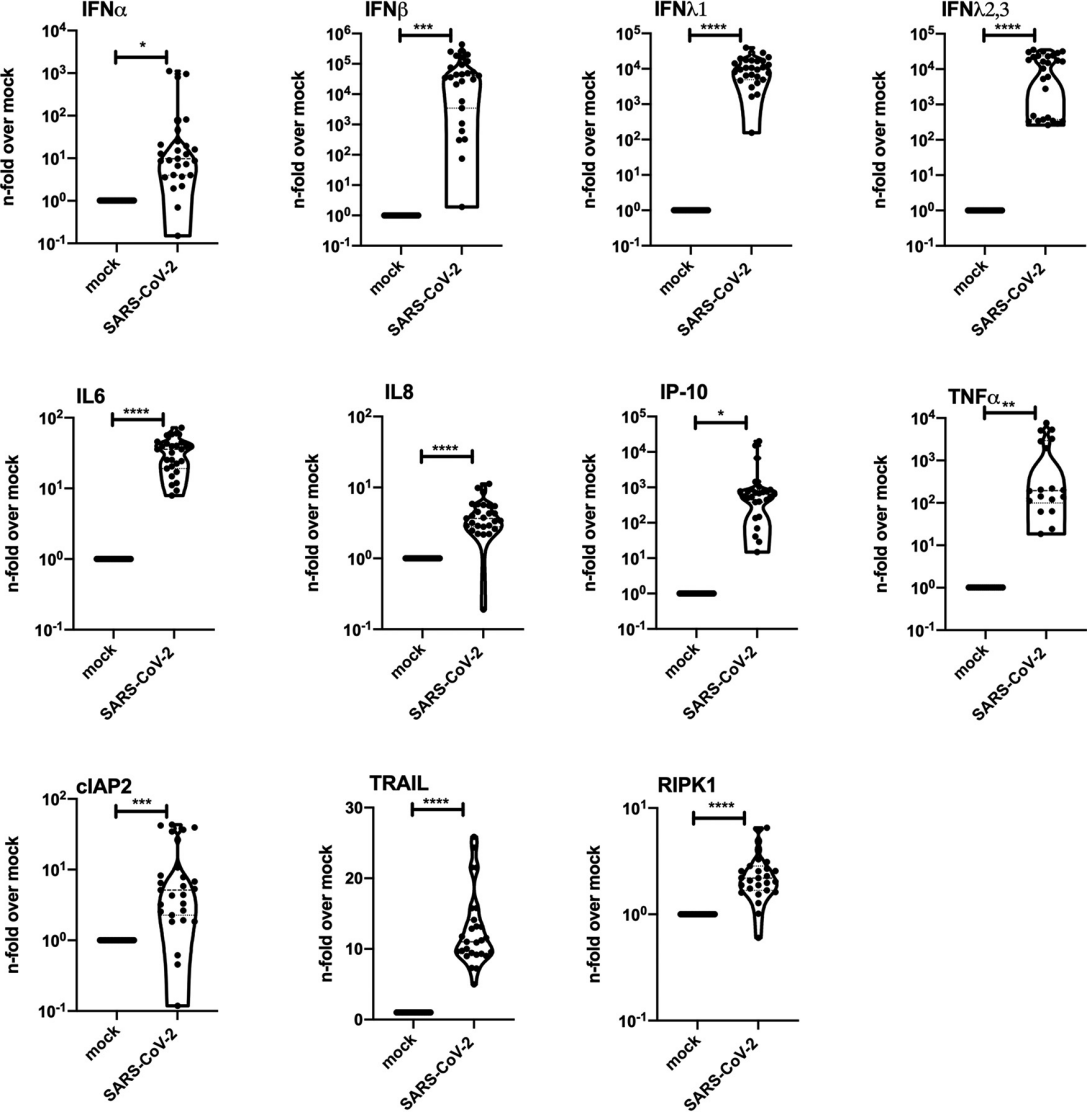
SARS-CoV-2 infection results in induction of antiviral and proinflammatory mRNA synthesis. Calu-3 cells were left uninfected (mock) or were infected with a SARS-CoV-2 patient isolate (5159, 5587, 5588) (MOI, 1). RNA lysates were performed 24 h p.i. Levels of IFNα, IFNβ, IFNλ1, IFNλ2,3, interleukin-6 (IL-6), IL-8, IP10 (interferon-gamma induced protein 10 kDa), tumor necrosis factor-alpha (TNF-α), cIAP2 (cellular inhibitor of apoptosis), TRAIL (TNF-related apoptosis inducing ligand), and RIPK1 (receptor-interacting serin/threonine-protein kinase) mRNA were measured for three patient isolates (5159, 5587, 5588) and two technical samples in 3 independent experiments. Means ± SD of three independent experiments are shown. Levels of mock-treated samples were arbitrarily set as 1. After normalization, two-tailed unpaired *t* tests were performed for comparison of mock-treated and SARS-CoV-2-infected samples (*, *P* < 0.05; **, *P* < 0.01; ***; *P* < 0.001; ****, *P* < 0.0001).

We further analyzed the host response to the infection by measuring the cytokine mRNA expression of Calu-3 cells. Many inflammatory cytokines were significantly increased compared to control cells 24 h postinfection (Fig. 3). These results reflect the high cytokine levels found in COVID-19 patients (16), indicating that infected epithelial cells might contribute to the disease development in severe COVID-19 cases. Since different cytokines and chemokines are involved in the infection process, a robust immune response has been associated with a severe clinical course (17). Our results clearly show that the epithelial cell line Calu-3 can be efficiently infected by SARS-CoV-2, propagates the virus, and responds to the viral infection with a strong cytokine release.

### SARS-CoV-2 infects epithelial cells within the human chip model, causing a strong IFN response.

**FIG 4:**
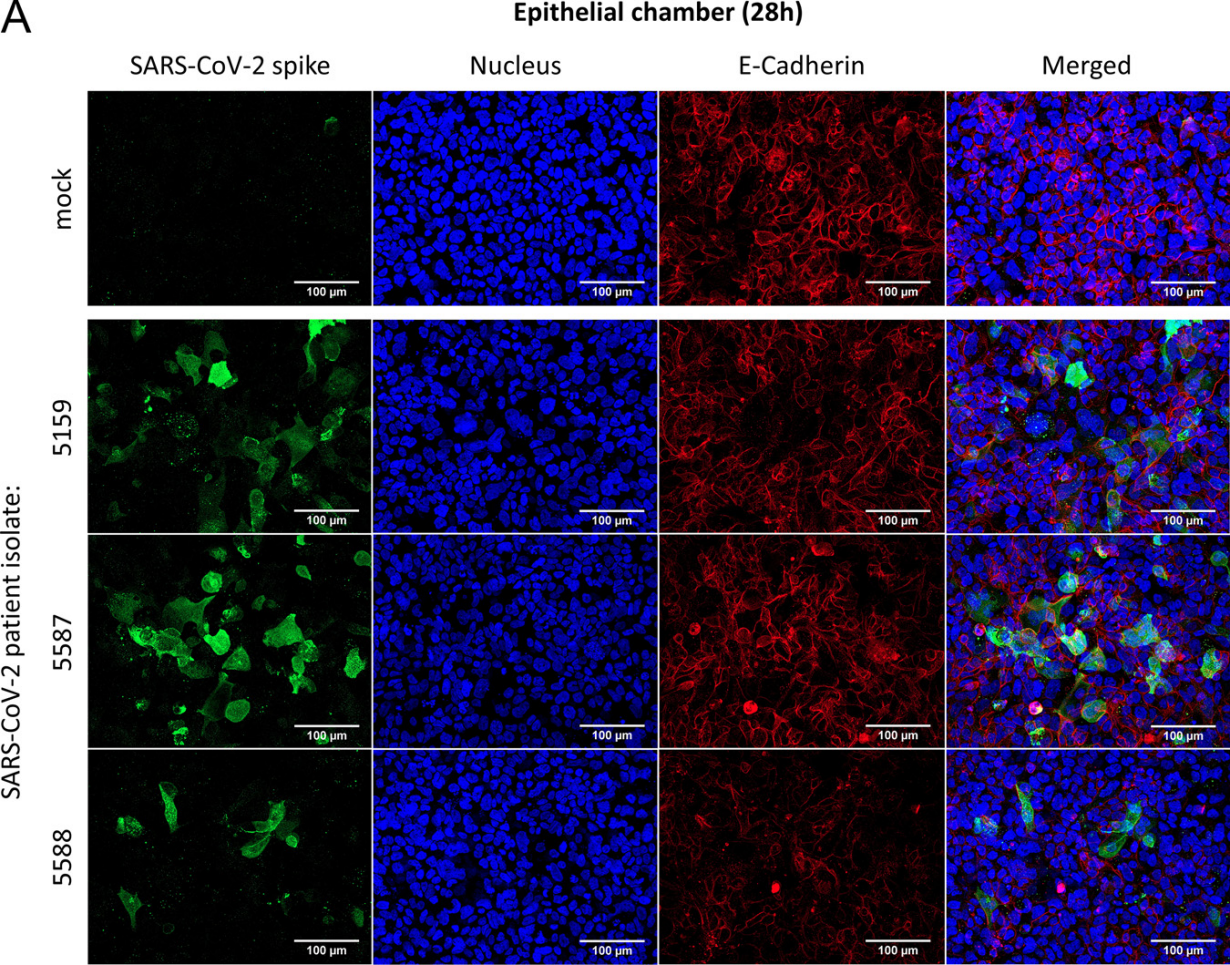
SARS-CoV-2 efficiently infects epithelial cells of the human chip model and provokes type I and III interferon production. The epithelial chamber of the chip model was left uninfected (mock) or infected with three different SARS-CoV-2 patient isolates (5159, 5587, 5588) (MOI, 1). (A and B) Immunofluorescence staining was performed 28 h p.i. and analyzed by immunofluorescence microscopy (Axio Observer.Z1; Zeiss); (A) the E-cadherin of the epithelial layer and (B) the VE-cadherin of the endothelial layer were visualized by an anti-E-cadherin-specific antibody or an anti-VE-cadherin antiserum, respectively, and a Cy5 goat anti-rabbit IgG (red). In panels A and B, the SARS-CoV-2 was visualized by detection of the spike protein via a spike-specific antibody and an Alexa Fluor 488-conjugated goat anti-mouse IgG (green). The nuclei were stained with Hoechst 33342 (blue). Scale bars represent 100 μm. (C) Production of antiviral cytokines derived from the epithelial side was determined by use of Legendplex panel (Biolegend, CA, USA). SARS-CoV-2-induced IFNβ, IFNλ1, and IFNλ2,3 release (pg ml^−1^) was measured. Means ± SD of three independent experiments each infected with another patient isolate (5159, 5587, 5588) are shown. After normalization, two-tailed unpaired *t* tests were performed for comparison of mock-treated and SARS-CoV-2-infected samples (**, *P* < 0.01).

In the next step, we modified our human chip model (9) by seeding Calu-3 cells on the epithelial side, primarily isolated HUVECs on the endothelial side, and integrated PBMCs to represent the immune response. However, macrophages that are mainly involved in inflammatory responses (18) do not show productive viral replication (19) (Fig. 2A to D). The applied system is ventilated and perfused and can be infected with SARS-CoV-2 via the epithelial side. Using the viral particles isolated from the three COVID-19 patients’ specimens, an infection by SARS-CoV-2 of the epithelial cells was proven, and the viral protein synthesis was visualized at 28 h postinfection (Fig. 4A). Accumulation of the SARS-CoV-2 spike protein was not yet visible at 8 h, but we were able to visualize the viral protein up to 40 h postinfection (Fig. S2A and C). However, SARS-CoV-2 spike protein was not visible in endothelial cells (Fig. 4B, Fig. S2B and D). In response to the infection, the epithelial cells reacted with a robust cytokine response, demonstrated by elevated interferon (IFN) levels in the cell culture supernatants of the epithelial side (Fig. 4C).

In general, the production of IFN is the most efficient way of fighting viral infections; e.g., secretion of type I IFN (IFN-α/β) exhibits direct antiviral effects by inhibiting viral replication (20) among many other interferon effects that promote the immune response to infection (21). Yet evasion strategies for different types of coronaviruses have been described. The viruses express factors and possess strategies to inhibit IFN induction/expression (22) or IFN signaling or to increase IFN resistance, which is reviewed by Kindler and Thiel (21). Consequently, SARS-CoV-2 is apparently able to cope with the interferon response of epithelial cells, which is reflected by our measurements, demonstrating efficient viral replication and persistence for up to 40 h despite a strong epithelial-mediated interferon response (Fig. 3 and 4, Fig. S2).

In contrast, we did not detect viral propagation and did not measure an interferon response at the endothelial side of the biochip (Fig. 4C). We could not visualize viral components within endothelial cells either at 8 h (Fig. S2B), 28 h (Fig. 4B), or 40 h postinfection (Fig. S2D), demonstrating that the viral particles do not productively infect endothelial cells in the human chip model. Additionally, endothelial mono-cell culture systems could not be infected by SARS-CoV-2 (Fig. 2A to D), confirming the cell-type specificity of the viral pathogens for lung epithelial cells. This is in line with *in vivo* studies that describe only a weak IFN response in the serum of COVID patients (23). In animal models with mouse-adapted SARS virus, a delayed onset of the IFN response resulting in immune dysregulation was described (24). The weak and delayed IFN levels in the serum are probably due to the host cell specificity of SARS-CoV-2, as lung epithelium represents the main infection focus, and endothelial cells are hardly infectible.

Taken together, these results indicate that endothelial cells are not the primary target cells of SARS-CoV-2, which is in agreement with previous studies (1, 25, 26). Further, it is in line with the observation that endothelial layers of the alveolar capillaries of deceased COVID-19 patients were still intact, but epithelial tissue was found to be seriously damaged (5). Although the mechanism is not explored, the increased proinflammatory cytokine release might cause an endothelial dysfunction. In addition, comorbidities such as obesity and diabetes might render endothelial cells more susceptible to being infected, as recently described (27, 28). There is clinical evidence for severe courses of COVID-19, in particular, when preexisting endothelial damage is suspected (29).

Further studies are required to elaborate the impact of the endothelial phenotype and the infection conditions at which these cells are targeted by SARS-CoV-2. In the model used in this study, the pneumotropic features of SARS-CoV-2 could be confirmed based on viral uptake and replication in epithelial cells accompanied by an interferon response described in previous studies. The viral particles were not transferred to the neighboring endothelial layer, although the cells were cocultivated in close proximity.

### SARS-CoV-2 disrupts the cellular barrier within the human chip model.

**FIG 5:**
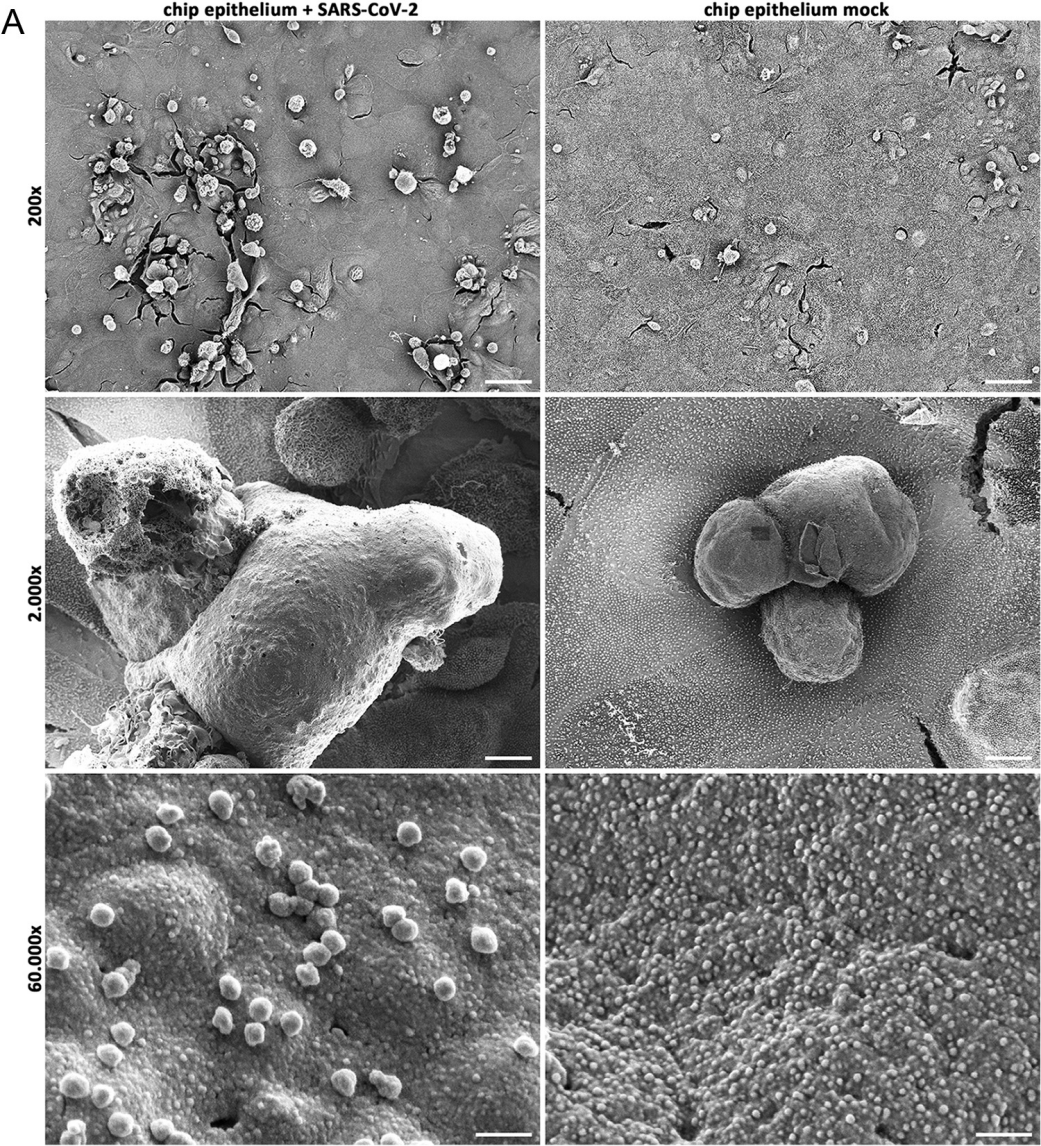
SARS-CoV-2 infection results in the disruption of barrier integrity in the human chip model. The epithelial side of the chip model was left uninfected (mock) or infected with the SARS-CoV-2 patient isolate (5159) (MOI, 1). (A and B) Scanning electron microscopy was performed 28 h p.i.; Overviews (top panel) of the (A) epithelial layer and (B) endothelial layer are depicted. Dead cells (middle panel) are focused. The surface of dead cells (lower panel) shows particles attached to the plasma membranes of the epithelial cells only. Scale bars represent 50 μm (×200 magnification), 5 μm (×2,000 magnification), and 200 nm (×60,000 magnification). (C) Supernatants of the epithelial and endothelial side of SARS-CoV-2-infected human chip models were used to perform LDH assays indicating cell membrane rupture at 8 h, 28 h, and 40 h p.i. (D) The barrier function of the human chip model was analyzed with a permeability assay of mock-infected and SARS-CoV-2-infected human chip models using FITC-dextran at 8 h and 28 h p.i. FITC-dextran was measured via the fluorescence intensity (excision, 488 nm; emission, 518 nm) and depicted as the permeability coefficient (*P_app_*), calculated according to *P_app_* (cm s^−1^) = (dQ/d*t*) (1/AC_o_). Results show significantly higher barrier permeability 28 h p.i. after SARS-CoV-2 infection. (E) Progeny virus titers were analyzed in the supernatants of the epithelial and endothelial layer with a standard plaque assay at 8 h, 28 h, and 40 h p.i. and indicated as plaque-forming units (PFU) ml^−1^. Shown are means (±SD) of three independent experiments each infected with a different patient isolate (5159, 5587, 5588). Statistical significance was analyzed by unpaired, two-tailed *t* test (*, *P* < 0.05; **, *P* < 0.01).

Next, we analyzed the barrier function of the epithelial-endothelial cell layers in our biochip model. Many clinical case reports and studies describe that critically ill COVID-19 patients develop severe lung destruction and a systemic sepsis-like syndrome (30, 31) that could be partly explained by a disrupted barrier function in the lung. From the immunofluorescence image (Fig. 4), we could observe some destruction of the epithelial or endothelial layer, in particular, 40 h postinfection (Fig. S2C and D). To better visualize the cell layers during the infection, we performed scanning electron microscopy (SEM) analysis of the surface structures 28 h postinfection. On the epithelial side, we found dead cells and remnants attached to the cell layer. Dying cells are identified by shrinking, balling, disruption of the plasma membrane, and the loss of microvilli, which can be observed in the top and middle panel of the infected cells but to a much lesser extent in mock-treated cells (Fig. 5A, top panel). At the surface of dead cells of the infected epithelial layer (Fig. 5A, bottom panel) large numbers of uniformly sized spherical particles attached to the cell membrane could be found. Based on their shape and size (83.4 ± 5.3 nm, *n* = 80) they can be considered to be SARS-CoV-2 virus particles.

It is well known that respiratory viral pathogens, such as the influenza virus (32), induce cell death (including apoptosis) in the respiratory epithelium. Previously, the 2003 SARS-CoV led to apoptotic cell death induced by membrane proteins via modulation of the Akt pathway (33). Additionally, prolonged stress of the endoplasmic reticulum (ER) was identified as a trigger for apoptosis (34). During the recent pandemic, it has been shown that the largest unique open reading frame (ORF) of the SARS-CoV-2 genome, ORF3a, is associated with a proapoptotic activity (35). These studies indicate that the induction of an apoptotic process in the course of SARS-CoV-2 infection is highly probable.

In contrast, on the endothelial side, we could not observe differences in the morphological appearance between SARS-CoV-2-infected and mock-treated cells. Here, the cell integrity of the cell layers appeared intact apart from shrinking artifacts due to the drying procedure (Fig. 5B). At high magnification (bottom panel), dead cell remnants show granular residues of the cytoplasm, indicating the loss of the plasma membrane, but no viral particles could be visualized.

To measure the epithelial and endothelial cell viability, we performed lactate dehydrogenase (LDH) assays on cells grown in the biochip (Fig. 5C). SARS-CoV-2 infection did not result in LDH release of epithelial or endothelial layers at 8 h postinfection. However, at 28 h postinfection, we observed in the infected epithelial layer a significantly enhanced release of LDH, whereas SARS-CoV-2 did not induce an enhanced LDH release in endothelial cells (Fig. 5C). However, after more extended infection periods (40 h; Fig. 5C), the barrier function on the endothelial side was also affected. These results suggest that even if the endothelial layer is not infected, the cell integrity gets disturbed, most likely by cytokines released by neighboring cells. In this respect, it is known that high cytokine/interferon levels can induce the disruption of the alveolar barrier function (36–38).

To further analyze the barrier integrity of the biochip system on a functional level, we performed permeability assays using fluorescein isothiocyanate (FITC)-dextran to investigate the endothelial and epithelial cell barrier integrity. We were able to show that SARS-CoV-2 infection did not affect tissue permeability at 8 h postinfection but resulted in significantly increased tissue permeability after 28 h postinfection (Fig. 5D). As a consequence of the disrupted barrier, we detected viral particles by performing plaque assays of the cell supernatants in the endothelial chamber in a time-dependent manner, with very low levels at 8 h postinfection and higher levels at 40 h postinfection (Fig. 5E). These results show that endothelial cells are affected by the viral infection at late time points and that the disturbed cell integrity results in translocation of the viral particles over the cellular barrier.

## DISCUSSION

**FIG 6:**
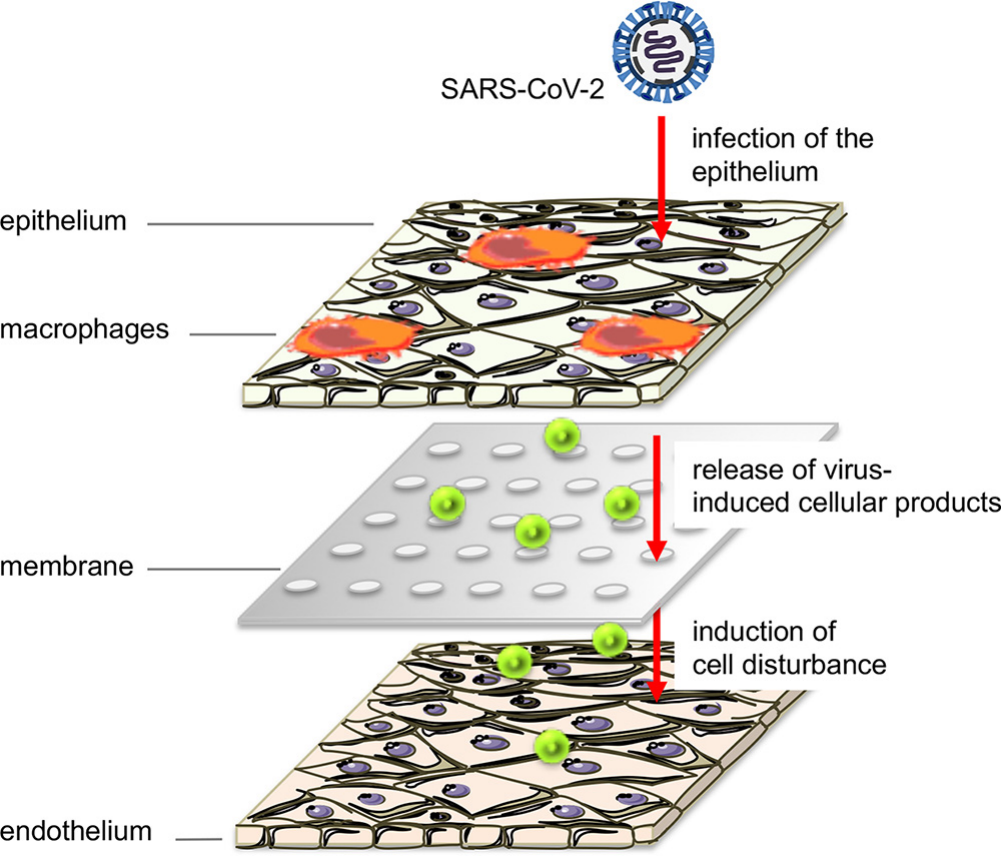
Schematic representation of a SARS-CoV-2-infected human chip model. SARS-CoV-2 productively infects the epithelium (Calu-3 cells) of the human chip model and produces progeny virus particles. Concomitantly, virus-induced cellular factors are released, affecting neighboring cells. Although the endothelial cells were cultured in close proximity to the infected epithelium, they were not infected by SARS-CoV-2. Nonetheless, endothelial cells become damaged, resulting in a decline in tissue barrier function. This figure was made with Servier Medical Art templates, which are licensed under a Creative Commons Attribution 3.0 Unported License.

In this article, we present an *in vitro* chip model based on human cells that can be efficiently infected by SARS-CoV-2 (Fig. 6). The epithelial cells of the model were prone to SARS-CoV-2 infection and propagated viral particles with high viral loads. These findings are in line with clinical observations that by far the highest viral burdens are measured in lung tissue (3). This phenomenon can be explained by the cell tropism of SARS-CoV-2 to airway cells that contribute to the high shedding of viral particles in the respiratory system and the high infectivity of patients via aerosols. Nevertheless, due to the systemic symptoms in severe COVID-19 patients, it has been discussed whether other cell types besides the airway epithelium are targeted by SARS-CoV-2, as well. In particular, vascular complications, such as thrombotic events (39), could result from the dissemination and propagation of viral particles in the endothelial system. In our model system, we could not confirm viral invasion into endothelial cells, although the cells were cultured in close proximity to the infected epithelium.

These results are in line with other recent publications, indicating that endothelial cells are resistant to SARS-CoV-2 infection and suggesting that vascular dysfunction is caused by factors and mediators released by adjacent infected cells (25, 26).

However, with increased time of infection (40 h), the endothelial cells become damaged, resulting in a decline in tissue barrier function. This effect is most likely mediated by the cytokine release of the infected neighboring epithelium. Cytokine release is known to disturb various cellular functions, such as protein biosynthesis and barrier integrity. Many studies reveal that most severe cases of SARS-CoV-2 infections are not only due to enhanced viral burden, but are to a large extent due to aberrant immune responses (38).

In our study, we present an infection model that could be further used to investigate several aspects of the SARS-CoV-2 infection. (i) At first, the cellular interaction can be analyzed in detail with increasing complexity. Here, the interaction between endothelial and epithelial cells and the role of different immune cells that can be integrated into the biochip could be elaborated. (ii) Another crucial aspect is preexisting damage, such as diabetic vascular changes or inflammatory foci that may promote a SARS-CoV-2 infection. These factors can be mimicked in the biochip model to investigate their impact on infection development. (iii) A third important issue relates to novel therapeutic agents. Antiviral and anti-inflammatory therapies can be tested in the biochip model to obtain initial results on their mode of action.

Consequently, our biochip model represents a valuable tool to study many aspects of SARS-CoV-2 infections.

## MATERIALS AND METHODS

### Virus isolation, propagation, and standard plaque assays.

For all experiments, we used SARS-CoV-2 isolated from respiratory specimens of three different patients (SARS-CoV-2/hu/Germany/Jena-vi005159/2020 [5159], SARS-CoV-2/hu/Germany/Jena-vi005587/2020 [5587], and SARS-CoV-2/hu/Germany/Jena-vi005588/2020 [5588]) (ethics approval of the Jena University Hospital, no. 2018-1263). For this, Vero-76 cells were washed 12 h after seeding and infected with 200 μl filtered patient sample (sterilized syringe filter; pore size, 0.2 μm) with the addition of Panserin 401 (PanBiotech, Germany). The cytopathic effect was detectable after 5 days. Then, the cells were further processed by freezing, centrifugation, and clear supernatant removal. Plaque purification was performed to achieve homogeneous viral stocks as follows: Vero-76 cells were infected with serial dilutions of virus isolates diluted in Eagle minimum essential medium (EMEM) for 60 min at 37°C and 5% CO_2_. The inoculum was exchanged with 2 ml EMEM/BA (medium with 0.2% bovine serum albumin [BSA]) supplemented with 0.9% agar (Oxoid, Wesel, Germany), 0.01% DEAE-Dextran (Pharmacia Biotech, Germany), and 0.2% NaHCO_3_ until plaque formation could be observed. The single viral plaques were marked by using inverse microscopy and used to infect confluent Vero-76 cell monolayers in T25 flasks. Cells were incubated at 37°C and 5% CO_2_ until pronounced cytopathic effects were visible. Then, cell cultures were frozen, and clear supernatants were collected. This plaque purification procedure was repeated again. Finally, virus stocks were generated and titrated using plaque assays. For this, Vero-76 cells were seeded in 6-well plates until a 90% confluence and infected with serial dilutions of the supernatants in phosphate-buffered saline (PBS)/BA (1 mM MgCl_2_, 0.9 mM CaCl, 0.2% BSA, 100 U ml^−1^ Pen/Strep) for 90 min at 37°C. After aspiration of the inoculum, cells were incubated with 2 ml MEM/BA (medium with 0.2% BSA) supplemented with 0.9% agar (Oxoid, Wesel, Germany), 0.01% DEAE-Dextran (Pharmacia Biotech, Germany), and 0.2% NaHCO_3_ at 37°C and 5% CO_2_ for 4 days. To visualize the plaques, the staining with crystal violet solution (0.2% crystal violet, 20% ethanol, 3.5% formaldehyde in water) was performed, and the number of infectious particles (PFU ml^−1^) was determined by counting.

### Sequencing and genome reconstruction.

Library preparation was performed according to the “nCoV-2019 sequencing protocol” (doi.org/10.17504/protocols.io.bdp7i5rn) from the ARTICnetwork (https://artic.network/ncov-2019). Briefly, viral RNA was isolated for SARS-CoV-2 virus strains 5159, 5587, and 5588 via the QIAmp viral RNA kit (Qiagen, Hilden, Germany) according to the manufacturer’s guide. The cDNA preparation was performed using SuperScript IV (Thermo Fisher), followed by a multiplex PCR to generate overlapping 400-nucleotide (nt) amplicons using version 3 of the primer set (https://github.com/artic-network/artic-ncov2019/tree/master/primer_schemes/nCoV-2019/V3). After PCR cleanup, library preparation was performed using the Ligation sequencing kit (LSK-109; Oxford Nanopore Technologies) and the native barcoding expansion (EXP-NBD104, native barcoding kit; Oxford Nanopore Technologies). Sequencing was performed on a MinION device using an R.9.4.1 flow cell (Oxford Nanopore Technologies). Base calling and genome reconstruction were performed using poreCov v.0.2 with the default settings (https://github.com/replikation/poreCov).

### Cell culture and virus infection.

For the virus propagation, we used Vero-76 cells, cultured in EMEM with HEPES, and 5 mM l-glutamine. Calu-3 cells were cultured in RPMI 1640 supplemented with 10% fetal calf serum (FCS). M199 was purchased from Lonza (Verviers, Belgium); fetal calf serum (FCS), human serum, and endothelial growth supplement were from Sigma (Taufkirchen, Germany).

For mono-cell culture studies with human monocyte-derived macrophages, monocytes were isolated from leukocyte concentrates obtained from freshly withdrawn peripheral blood of healthy male and female adult human donors (18 to 65 years old), which were provided by the Institute of Transfusion Medicine at the University Hospital Jena, Germany. The experimental protocol was approved by the ethics committee of the University Hospital Jena. All methods were performed in accordance with the relevant guidelines and regulations. Peripheral blood mononuclear cells (PBMC) were separated using dextran sedimentation, followed by centrifugation on lymphocyte separation medium (Histopaque-1077; Sigma-Aldrich, St. Louis, MO, USA). PBMC were seeded in RPMI 1640 (Sigma-Aldrich) containing 10% (vol/vol) heat-inactivated fetal calf serum (FCS), 100 U ml^−1^ penicillin, and 100 μg ml^−1^ streptomycin (Biochrom/Merck, Berlin, Germany) in cell culture flasks for 1.5 h at 37°C and 5% CO_2_ for adherence of monocytes. For differentiation of monocytes to macrophages, monocytes were incubated with 20 ng ml^−1^ granulocyte-macrophage colony-stimulating factor (GM-CSF; Peprotech, Hamburg, Germany) for 6 days in RPMI 1640 supplemented with 10% fetal calf serum, 2 mM glutamine (Biochrom/Merck), and penicillin/streptomycin.

For the integration of PBMCs into the human chip model, cells were isolated and cultivated as previously described (9). Human umbilical vein endothelial cells (HUVECs) were isolated from anonymously acquired human umbilical cords according to the Declaration of Helsinki, “Ethical Principles for Medical Research Involving Human Subjects” (1964). After the cord veins were rinsed with 0.9% NaCl, endothelial cells were detached with collagenase (0.01%, 3 min at 37°C), suspended in M199/10% FCS, washed once (500 × *g*, 6 min), and seeded on a cell culture flask coated with 0.2% gelatin. Then, 24 h later, full growth medium was added (M199, 17.5% FCS, 2.5% human serum, 7.5 μg ml^−1^ endothelial mitogen, 7.5 U ml^−1^ heparin, 680 μM glutamine, 100 μM vitamin C, 100 U ml^−1^ penicillin, 100 μg ml^−1^ streptomycin). HUVECs from the second passage were seeded on 30-mm dishes or on 90-mm dishes at a density of 27,500 cells/cm^2^. Experiments were performed 72 h after seeding. For the cultivation of the human chip model, we used Calu-3 cells and macrophages at the epithelial side and HUVECs at the endothelial side. The multiorgan tissue flow (MOTiF) biochips were manufactured and obtained from microfluidic ChipShop GmbH (Jena, Germany), as explained previously (9).

The *in vitro* infection of the cell lines was performed as follows: cells were washed with PBS and either left uninfected (mock) or infected with SARS-CoV-2 with a multiplicity of infection (MOI) of 1 for 120 min in medium (EMEM with HEPES modification and 5 mM l-glutamine for Vero-76 cells, RPMI 1640 for Calu-3 cells, RPMI 1640 with GM-CSF and penicillin/streptomycin for macrophages, and endothelial cell medium for HUVECs) supplemented with 10% FCS. Subsequently, supernatants were removed, and cells were supplemented with fresh medium containing 10% FCS and further incubated at 37°C and 5% CO_2_.

For the infection of the human chip model, cells were washed with PBS once, followed by treatment with PBS (mock) at 37°C and RPMI (0.2% autologous human serum, 1 mM MgCl_2_, 0.9 mM CaCl_2_) or infection with SARS-CoV-2 (1 MOI). After 90 min, incubation cells were washed and supplemented with medium. Afterward, cells were incubated for the indicated times at 37°C and 5% CO_2_.

### Transmission electron microscopy.

Confluent monolayers of Vero-76 cells or Calu-3 cells (9-cm petri dishes) were infected with SARS-CoV-2 (isolate 5159) using an MOI of 1. After 24 h, supernatants were removed, and samples were fixed with freshly prepared modified Karnovsky fixative consisting of 4% wt/vol paraformaldehyde and 2.5% vol/vol glutaraldehyde in 0.1 M sodium cacodylate buffer, pH 7.4, for 1 h at room temperature. After washing 3 times for 15 min each with 0.1 M sodium cacodylate buffer (pH 7.4), the cells were postfixed with 2% wt/vol osmium tetroxide for 1 h at room temperature. Subsequently, the cells were rewashed with 0.1 M sodium cacodylate buffer (pH 7.4), thoroughly scraped off the petri dishes, and pelleted by centrifuging at 600 × *g* for 10 min. During the following dehydration in ascending ethanol series, poststaining with 1% wt/vol uranyl acetate was performed. Afterward, the pellets were embedded in epoxy resin (Araldite) and ultrathin-sectioned (70 nm) using a Leica Ultracut S instrument (Leica, Wetzlar, Germany). Finally, the sections were mounted on filmed Cu grids, poststained with lead citrate, and studied in a transmission electron microscope (EM 900; Zeiss, Oberkochen, Germany) at 80 kV and magnifications of ×3,000 to ×85,000. For image recording, a 2K slow-scan charge-coupled device (CCD) camera (TRS, Moorenweis, Germany) was used.

### Immunofluorescence microscopy.

Membranes of the human chip model were fixed for at least 30 min with 4% paraformaldehyde at 37°C and permeabilized with 0.1% saponin buffer for 1 h at room temperature. For the human chip model, the membrane was removed from the chip after fixation and before permeabilization and cut in half to analyze either the epithelial or the endothelial side. Infection by SARS-CoV-2 was visualized using mouse anti-SARS-CoV-2 spike (GeneTex; no. GTX632604) IgG monoclonal, primary antibodies and Alexa Fluor goat anti-mouse IgG polyclonal antibodies (Dianova; no. 115-545-146). The nuclei were stained with bisBenzimide H 33342 trihydrochloride (Hoechst 33342) (Merck; no. 14533). Rabbit anti-E-cadherin IgG monoclonal (Cell Signaling; no. 3195S) or rabbit anti-VE-cadherin polyclonal, primary antibodies (Cell Signaling; no. 2158S) and Cy5 goat anti-rabbit IgG polyclonal antibodies (Dianova; no. 111-175-144) were used to detect cell borders of Calu-3 or HUVEC cells, respectively, on the membrane of the human chip model. Primary antibodies were added 1:100, overnight at 4°C. Afterward, secondary antibodies and Hoechst 33342 were added 1:100 and 1:1,000 for 1 h, at room temperature and in the dark. Cells and membranes were mounted with fluorescence mounting medium (Dako; no. S3023).

Images were acquired using an Axio Observer.Z1 microscope (Zeiss) with Plan Apochromat 20×/0.8 objective (Zeiss), ApoTome.2 (Zeiss), and Axiocam 503 mono (Zeiss) and the software Zen 2.6 (blue edition; Zeiss). Apotome defolding with phase error correction and deconvolution was done with the software Zen 2.6 as well. Fiji V 1.52b (ImageJ) was used for further image processing, including Z-stack merging with maximum intensity projection and gamma correction. Parameters were kept the same for all pictures which were compared with each other.

### Scanning electron microscopy.

The fixation of the cells was performed inside the human chip model by using the same fixative as for TEM for 60 min at room temperature as described previously (9, 40, 41). Afterward, the chips were rinsed three times with fresh cacodylate buffer for 10 min each, and the membranes were cut out. After postfixation with 2% wt/vol osmium tetroxide for 1 h, the samples were dehydrated in ascending ethanol concentrations (30, 50, 70, 90, and 100%) for 15 min each. Subsequently, the samples were critical-point dried using liquid CO_2_ and sputter coated with gold (thickness, approximately 2 nm) using a CCU-010 sputter coater (Safematic GmbH, Zizers, Switzerland). The specimens were investigated with a field emission SEM LEO-1530 Gemini (Carl Zeiss NTS GmbH, Oberkochen, Germany).

### Western blot analysis.

For Western blotting, cells were lysed with Triton lysis buffer (TLB; 20 mM Tris‐HCl, pH 7.4; 137 mM NaCl; 10% glycerol; 1% Triton X‐100; 2 mM EDTA; 50 mM sodium glycerophosphate, 20 mM sodium pyrophosphate; 5 μg ml^−1^ aprotinin; 5 μg ml^−1^ leupeptin; 1 mM sodium vanadate and 5 mM benzamidine) for 30 min. Cell lysates were cleared by centrifugation, supplemented with 5× Lämmli buffer (10% SDS, 50% glycerol, 25% 2-mercaptoethanol, 0.02% bromophenol blue, 312 mM Tris, 6.8 pH) (diluted 1:5), boiled for 10 min (95°C), and subjected to SDS‐PAGE and subsequent blotting. For the detection of SARS-CoV-2 spike protein, a rabbit polyclonal anti-SARS-CoV-2 spike S2 antibody (Sino Biological; no. 40590-T62) was used. Equal protein load was verified by use of a mouse monoclonal anti-ERK2 antibody (Santa Cruz; no. sc1647).

### Lactate dehydrogenase cytotoxicity assay.

Cell cytotoxicity was determined with a CyQUANT lactate dehydrogenase (LDH) cytotoxicity assay kit (Invitrogen/Thermo Fisher Scientific, Waltham, USA) according to the manufacturer’s instructions. Cells were infected as previously described. After infection, 25 μl of the supernatant was transferred in technical duplicates to a 96-well plate and mixed with 25 μl of the LDH cytotoxicity assay reagent. The plate was incubated at 37°C for 30 min. Stop solution (25 μl) was added, and the optical density at 492 nm (OD_492nm_) was directly measured using a Tecan Spectrafluor plate reader (Tecan Group Ltd., Maennedorf, Switzerland). The OD_620nm_ was subtracted to correct for background signal.

### Permeability assay.

To test the permeability of the epithelial and endothelial barriers, 1 mg ml^−1^ of 3- to 5-kDa fluorescein isothiocyanate (FITC)-dextran (Sigma-Aldrich, Germany) in phenol-red free DMEM/F12 medium (Sigma-Aldrich, Germany) was injected into the upper chamber of the chip. The lower chamber contained only phenol red free DMEM/F12. The alveolus model was incubated for 60 min under static conditions. Afterwards, the media from the lower and upper chambers were collected, and the fluorescence intensity (excision, 488 nm; emission, 518 nm) was measured in a 96-well *μ*Clear black plate (Greiner BioOne, Frickenhausen, Germany) using a FLUOStar Omega microplate reader (BMG Labtech GmbH, Ortenberg, Germany). The permeability coefficient (*P_app_*) was calculated according to *P_app_* (cm s^−1^) = (dQ/d*t*) (1/*A*C_o_). For this, dQ/d*t* represents the steady-state flux (g s^−1^), *A* represents the culture surface area (cm^2^), and C_o_ represents the initial concentration (mg ml^−1^) (42).

### Detection of mRNA-expression by using qRT-PCR.

For RNA isolation, cells were lysed with 350 μl RLT lysis buffer and detached from the plate using a rubber cell scraper. RNA isolation was performed using the RNeasy minikit (Qiagen, Hilden, Germany) according to the manufacturer’s protocol. RNA concentration was measured using the NanoDrop spectrophotometer ND-1000 (Peqlab/VWR, Radnor, USA).

For cDNA synthesis, the QuantiNova reverse transcription kit (Qiagen, Venlo, Netherlands) was used. RNA was thawed on ice, and 400 ng RNA was diluted in RNase-free water to a volume of 13 μl. Then, 2 μl gDNA removal mix was added to the diluted RNA, followed by incubation at 45°C for 2 min. After the samples were incubated for at least 1 min on ice, 5 μl of RT master mix (containing 4 μl reverse transcription mix and 1 μl reverse transcription enzyme per sample) was added. The resulting mixture was incubated for 3 min at 25°C, followed by incubation at 45°C for 10 min and an inactivation step at 85°C for 5 min. The cDNA was either directly used for the subsequent experiments or stored at −20°C.

The qRT-PCRs were performed using the QuantiNova SYBR green PCR kit (Qiagen, Venlo, Netherlands). Then, 1 μl cDNA was added to 19 μl master mix (containing 10 μl SYBR green, 1.5 μl forward primer [10 μM], 1.5 μl reverse primer [10 μM], and 6 μl RNase-free double-distilled water [ddH_2_O] per sample; for primer sequences, see Table S1), and the qRT-PCR was started using the following cycle conditions: 95°C for 2 min, followed by 40 cycles of 95°C for 5 sec and 60°C for 10 sec. The qRT-PCR cycle was ended by a stepwise temperature increase from 60°C to 95°C (1°C every 5 sec).

### Detection of SARS-CoV-2 by using qRT-PCR.

For the determination of SARS-CoV-2, we used the QIAamp viral RNA minikit (Qiagen, Hilden, Germany) according to manufacturer’s guide. This was followed by a qRT-PCR purchased by RIDAgene (r-biopharm, Darmstadt, Germany) on Rotor-Gene Q (Qiagen, Hilden, Germany) to detect the E-gene of SARS-CoV-2. The RNA standard curve, prepared from the positive control of the RIDAgene (r-biopharm, Darmstadt, Germany) kit, cycle conditions were set as follows: 10 min at 58°C, 1 min at 95°C, and 45 cycles of 95°C for 15 sec and 60°C for 30 sec.

### Statistical analysis.

Statistical analysis was performed using Prism 8 (GraphPad Software). Statistical methods are described in the figure legends.

### Data availability.

The newly determined sequences are available in GenBank under accession numbers MW633322 to MW633324.

## Supplementary Material

Supplemental file 1

Supplemental file 2

## References

[1] Bar-On YM, Flamholz A, Phillips R, Milo R. 2020. SARS-CoV-2 (COVID-19) by the numbers. Elife 9:e57309. 10.7554/eLife.57309. 32228860 PMC7224694

[2] George PM, Wells AU, Jenkins RG. 2020. Pulmonary fibrosis and COVID-19: the potential role for antifibrotic therapy. Lancet Respir Med 8:807–815. 10.1016/S2213-2600(20)30225-3. 32422178 PMC7228727

[3] Carsana L, Sonzogni A, Nasr A, Rossi RS, Pellegrinelli A, Zerbi P, Rech R, Colombo R, Antinori S, Corbellino M, Galli M, Catena E, Tosoni A, Gianatti A, Nebuloni M. 2020. Pulmonary post-mortem findings in a series of COVID-19 cases from northern Italy: a two-centre descriptive study. Lancet Infectious Diseases 20:1135–1140. 10.1016/S1473-3099(20)30434-5. 32526193 PMC7279758

[4] Wichmann D, Sperhake J-P, Lütgehetmann M, Steurer S, Edler C, Heinemann A, Heinrich F, Mushumba H, Kniep I, Schröder AS, Burdelski C, de Heer G, Nierhaus A, Frings D, Pfefferle S, Becker H, Bredereke-Wiedling H, de Weerth A, Paschen H-R, Sheikhzadeh-Eggers S, Stang A, Schmiedel S, Bokemeyer C, Addo MM, Aepfelbacher M, Püschel K, Kluge S. 2020. Autopsy findings and venous thromboembolism in patients with COVID-19. Ann Intern Med 173:268–277. 10.7326/M20-2003. 32374815 PMC7240772

[5] Deinhardt-Emmer S, Wittschieber D, Sanft J, Kleemann S, Elschner S, Haupt KF, Vau V, Häring C, Rödel J, Henke A, Ehrhardt C, Bauer M, Philipp M, Gaßler N, Nietzsche S, Löffler B, Mall G. 2020. Early postmortem mapping of SARS-CoV-2 RNA in patients with COVID-19 and correlation to tissue damage. bioRxiv 10.1101/2020.07.01.182550:2020.07.01.182550. PMC8009677 33781385

[6] Becker RC. 2020. COVID-19 update: Covid-19-associated coagulopathy. J Thromb Thrombolysis 50:54–67. 10.1007/s11239-020-02134-3. 32415579 PMC7225095

[7] Hoffmann M, Kleine-Weber H, Schroeder S, Krüger N, Herrler T, Erichsen S, Schiergens TS, Herrler G, Wu NH, Nitsche A, Müller MA, Drosten C, Pöhlmann S. 2020. SARS-CoV-2 cell entry depends on ACE2 and TMPRSS2 and is blocked by a clinically proven protease inhibitor. Cell 181:271–280.e8. 10.1016/j.cell.2020.02.052. 32142651 PMC7102627

[8] Bestle D, Heindl MR, Limburg H, Van Lam van T, Pilgram O, Moulton H, Stein DA, Hardes K, Eickmann M, Dolnik O, Rohde C, Klenk HD, Garten W, Steinmetzer T, Böttcher-Friebertshäuser E. 2020. TMPRSS2 and furin are both essential for proteolytic activation of SARS-CoV-2 in human airway cells. Life Sci Alliance 3:e202000786. 10.26508/lsa.202000786. 32703818 PMC7383062

[9] Deinhardt-Emmer S, Rennert K, Schicke E, Cseresnyés Z, Windolph M, Nietzsche S, Heller R, Siwczak F, Haupt KF, Carlstedt S, Schacke M, Figge MT, Ehrhardt C, Löffler B, Mosig AS. 2020. Co-infection with Staphylococcus aureus after primary influenza virus infection leads to damage of the endothelium in a human alveolus-on-a-chip model. Biofabrication 12:025012. 10.1088/1758-5090/ab7073. 31994489

[10] Gorbalenya AE, Baker SC, Baric RS, de Groot RJ, Drosten C, Gulyaeva AA, Haagmans BL, Lauber C, Leontovich AM, Neuman BW, Penzar D, Perlman S, Poon LLM, Samborskiy DV, Sidorov IA, Sola I, Ziebuhr J, Coronaviridae Study Group of the International Committee on Taxonomy of V. 2020. The species Severe acute respiratory syndrome-related coronavirus: classifying 2019-nCoV and naming it SARS-CoV-2. Nat Microbiol 5:536–544. 10.1038/s41564-020-0695-z. 32123347 PMC7095448

[11] Tang X, Wu C, Li X, Song Y, Yao X, Wu X, Duan Y, Zhang H, Wang Y, Qian Z, Cui J, Lu J. 2020. On the origin and continuing evolution of SARS-CoV-2. National Science Rev 7:1012–1023. 10.1093/nsr/nwaa036. PMC7107875 34676127

[12] Rambaut A, Holmes EC, O’Toole Á, Hill V, McCrone JT, Ruis C, Du Plessis L, Pybus OG. 2020. A dynamic nomenclature proposal for SARS-CoV-2 lineages to assist genomic epidemiology. Nat Microbiol 5:1403–1407. 10.1038/s41564-020-0770-5. 32669681 PMC7610519

[13] Shang J, Wan Y, Luo C, Ye G, Geng Q, Auerbach A, Li F. 2020. Cell entry mechanisms of SARS-CoV-2. Proc Natl Acad Sci U S A 117:11727–11734. 10.1073/pnas.2003138117. 32376634 PMC7260975

[14] Stertz S, Reichelt M, Spiegel M, Kuri T, Martínez-Sobrido L, García-Sastre A, Weber F, Kochs G. 2007. The intracellular sites of early replication and budding of SARS-coronavirus. Virology 361:304–315. 10.1016/j.virol.2006.11.027. 17210170 PMC7103305

[15] Ogando NS, Dalebout TJ, Zevenhoven-Dobbe JC, Limpens RW, van der Meer Y, Caly L, Druce J, de Vries JJC, Kikkert M, Bárcena M, Sidorov I, Snijder EJ. 2020. SARS-coronavirus-2 replication in Vero E6 cells: replication kinetics, rapid adaptation and cytopathology. bioRxiv 10.1101/2020.04.20.049924:2020.04.20.049924. PMC7654748 32568027

[16] Costela-Ruiz VJ, Illescas-Montes R, Puerta-Puerta JM, Ruiz C, Melguizo-Rodríguez L. 2020. SARS-CoV-2 infection: the role of cytokines in COVID-19 disease. Cytokine Growth Factor Rev 54:62–75. 10.1016/j.cytogfr.2020.06.001. 32513566 PMC7265853

[17] Coperchini F, Chiovato L, Croce L, Magri F, Rotondi M. 2020. The cytokine storm in COVID-19: An overview of the involvement of the chemokine/chemokine-receptor system. Cytokine Growth Factor Rev 53:25–32. 10.1016/j.cytogfr.2020.05.003. 32446778 PMC7211650

[18] Kumar S, Nyodu R, Maurya VK, Saxena SK. 2020. Host immune response and immunobiology of human SARS-CoV-2 infection, p 43–53. *In* Saxena S (ed), Coronavirus disease 2019 (COVID-19). medical virology: from pathogenesis to disease control. 10.1007/978-981-15-4814-7_5:43-53. Springer, Singapore.

[19] Yip MS, Leung NHL, Cheung CY, Li PH, Lee HHY, Daëron M, Peiris JSM, Bruzzone R, Jaume M. 2014. Antibody-dependent infection of human macrophages by severe acute respiratory syndrome coronavirus. Virol J 11:82–82. 10.1186/1743-422X-11-82. 24885320 PMC4018502

[20] Thiel V, Weber F. 2008. Interferon and cytokine responses to SARS-coronavirus infection. Cytokine Growth Factor Rev 19:121–132. 10.1016/j.cytogfr.2008.01.001. 18321765 PMC7108449

[21] Kindler E, Thiel V. 2016. SARS-CoV and IFN: too little, too late. Cell Host Microbe 19:139–141. 10.1016/j.chom.2016.01.012. 26867172 PMC7104995

[22] Thoms M, Buschauer R, Ameismeier M, Koepke L, Denk T, Hirschenberger M, Kratzat H, Hayn M, Mackens-Kiani T, Cheng J, Straub JH, Stürzel CM, Fröhlich T, Berninghausen O, Becker T, Kirchhoff F, Sparrer KMJ, Beckmann R. 2020. Structural basis for translational shutdown and immune evasion by the Nsp1 protein of SARS-CoV-2. Science 369:1249–1255. 10.1126/science.abc8665. 32680882 PMC7402621

[23] Hu B, Huang S, Yin L. 2021. The cytokine storm and COVID-19. J Med Virol 93:250–256. 10.1002/jmv.26232. 32592501 PMC7361342

[24] Channappanavar R, Fehr AR, Vijay R, Mack M, Zhao J, Meyerholz DK, Perlman S. 2016. Dysregulated type I interferon and inflammatory monocyte-macrophage responses cause lethal pneumonia in SARS-CoV-infected mice. Cell Host Microbe 19:181–193. 10.1016/j.chom.2016.01.007. 26867177 PMC4752723

[25] Ahmetaj-Shala B, Peacock TP, Baillon L, Swann OC, Gashaw H, Barclay WS, Mitchell JA. 2020. Resistance of endothelial cells to SARS-CoV-2 infection. bioRxiv 10.1101/2020.11.08.372581:2020.11.08.372581. PMC12724323 41347787

[26] Michalick L, Weidenfeld S, Grimmer B, Fatykhova D, Solymosi PD, Behrens F, Dohmen M, Brack MC, Schulz S, Thomasch E, Simmons S, Müller-Redetzky H, Suttorp N, Kurth F, Neudecker J, Toennies M, Bauer TT, Eggeling S, Corman VM, Hocke AC, Witzenrath M, Hippenstiel S, Kuebler WM. 2020. Plasma mediators in patients with severe COVID-19 cause lung endothelial barrier failure. Eur Resp J 10.1183/13993003.02384-2020:2002384. PMC7651836 33154030

[27] Huertas A, Montani D, Savale L, Pichon J, Tu L, Parent F, Guignabert C, Humbert M. 2020. Endothelial cell dysfunction: a major player in SARS-CoV-2 infection (COVID-19)? Eur Respir J 56:2001634. 10.1183/13993003.01634-2020. 32554538 PMC7301835

[28] Pons S, Fodil S, Azoulay E, Zafrani L. 2020. The vascular endothelium: the cornerstone of organ dysfunction in severe SARS-CoV-2 infection. Crit Care 24:353–353. 10.1186/s13054-020-03062-7. 32546188 PMC7296907

[29] Varga Z, Flammer AJ, Steiger P, Haberecker M, Andermatt R, Zinkernagel AS, Mehra MR, Schuepbach RA, Ruschitzka F, Moch H. 2020. Endothelial cell infection and endotheliitis in COVID-19. Lancet 395:1417–1418. 10.1016/S0140-6736(20)30937-5. 32325026 PMC7172722

[30] Gao YM, Xu G, Wang B, Liu BC. 2020. Cytokine storm syndrome in coronavirus disease 2019: a narrative review. J Intern Med 289:147–161. 10.1111/joim.13144. 32696489 PMC7404514

[31] Ackermann M, Verleden SE, Kuehnel M, Haverich A, Welte T, Laenger F, Vanstapel A, Werlein C, Stark H, Tzankov A, Li WW, Li VW, Mentzer SJ, Jonigk D. 2020. Pulmonary vascular endothelialitis, thrombosis, and angiogenesis in Covid-19. N Engl J Med 383:120–128. 10.1056/NEJMoa2015432. 32437596 PMC7412750

[32] Atkin-Smith GK, Duan M, Chen W, Poon IKH. 2018. The induction and consequences of Influenza A virus-induced cell death. Cell Death Dis 9:1002. 10.1038/s41419-018-1035-6. 30254192 PMC6156503

[33] Chan C-M, Ma C-W, Chan W-Y, Chan HYE. 2007. The SARS-coronavirus membrane protein induces apoptosis through modulating the Akt survival pathway. Arch Biochem Biophys 459:197–207. 10.1016/j.abb.2007.01.012. 17306213 PMC7094499

[34] Fung TS, Liu DX. 2014. Coronavirus infection, ER stress, apoptosis and innate immunity. Front Microbiol 5:296. 10.3389/fmicb.2014.00296. 24987391 PMC4060729

[35] Ren Y, Shu T, Wu D, Mu J, Wang C, Huang M, Han Y, Zhang X-Y, Zhou W, Qiu Y, Zhou X. 2020. The ORF3a protein of SARS-CoV-2 induces apoptosis in cells. Cell Mol Immunol 17:881–883. 10.1038/s41423-020-0485-9. 32555321 PMC7301057

[36] Pelaia C, Tinello C, Vatrella A, De Sarro G, Pelaia G. 2020. Lung under attack by COVID-19-induced cytokine storm: pathogenic mechanisms and therapeutic implications. Ther Adv Respir Dis 14:1753466620933508. 10.1177/1753466620933508. 32539627 PMC7298425

[37] Gustafson D, Raju S, Wu R, Ching C, Veitch S, Rathnakumar K, Boudreau E, Howe KL, Fish JE. 2020. Overcoming barriers: the endothelium as a linchpin of coronavirus disease 2019 pathogenesis? Arterioscler Thromb Vasc Biol 40:1818–1829. 10.1161/ATVBAHA.120.314558. 32510978 PMC7370857

[38] Broggi A, Ghosh S, Sposito B, Spreafico R, Balzarini F, Lo Cascio A, Clementi N, De Santis M, Mancini N, Granucci F, Zanoni I. 2020. Type III interferons disrupt the lung epithelial barrier upon viral recognition. Science 369:706–712. 10.1126/science.abc3545. 32527925 PMC7292499

[39] Helms J, Tacquard C, Severac F, Leonard-Lorant I, Ohana M, Delabranche X, Merdji H, Clere-Jehl R, Schenck M, Fagot Gandet F, Fafi-Kremer S, Castelain V, Schneider F, Grunebaum L, Anglés-Cano E, Sattler L, Mertes P-M, Meziani F, Group CT, CRICS TRIGGERSEP Group (Clinical Research in Intensive Care and Sepsis Trial Group for Global Evaluation and Research in Sepsis). 2020. High risk of thrombosis in patients with severe SARS-CoV-2 infection: a multicenter prospective cohort study. Intensive Care Med 46:1089–1098. 10.1007/s00134-020-06062-x. 32367170 PMC7197634

[40] Rennert K, Steinborn S, Gröger M, Ungerböck B, Jank AM, Ehgartner J, Nietzsche S, Dinger J, Kiehntopf M, Funke H, Peters FT, Lupp A, Gärtner C, Mayr T, Bauer M, Huber O, Mosig AS. 2015. A microfluidically perfused three dimensional human liver model. Biomaterials 71:119–131. 10.1016/j.biomaterials.2015.08.043. 26322723

[41] Maurer M, Gresnigt MS, Last A, Wollny T, Berlinghof F, Pospich R, Cseresnyes Z, Medyukhina A, Graf K, Gröger M, Raasch M, Siwczak F, Nietzsche S, Jacobsen ID, Figge MT, Hube B, Huber O, Mosig AS. 2019. A three-dimensional immunocompetent intestine-on-chip model as in vitro platform for functional and microbial interaction studies. Biomaterials 220:119396. 10.1016/j.biomaterials.2019.119396. 31398556

[42] Thomas L, Rao Z, Gerstmeier J, Raasch M, Weinigel C, Rummler S, Menche D, Muller R, Pergola C, Mosig A, Werz O. 2017. Selective upregulation of TNFalpha expression in classically-activated human monocyte-derived macrophages (M1) through pharmacological interference with V-ATPase. Biochem Pharmacol 130:71–82. 10.1016/j.bcp.2017.02.004. 28189727

