# Supplementary material for: SARS-CoV-2 Causes Severe Epithelial Inflammation and Barrier Dysfunction
Source: J Virol. 2021 Apr 26;95(10):e00110-21. doi: 10.1128/JVI.00110-21 (PMC8139673; doi:10.1128/JVI.00110-21)
Supplement: Supplemental file 1 [file JVI.00110-21-s0001.pdf]

STAR ★ METHODS

KEY RESOURCES TABLES

REAGENT or RESOURCE	SOURCE	IDENTIFIER
**Antibodies**		
SARS-CoV-2 spike, mouse	GeneTex	GTX632604
E-Cadherin, rabbit	Cell Signaling	3195S
VE-Cadherin, rabbit	SinoBiological	2158S
ERK2, mouse	Santa Cruz	sc-1647
SARS-CoV-2/2019-nCoV Spike/S2 Rabbit Antibody	Sino Biological	40590-T62
Goat anti-Mouse IgG (H+L) Secondary Antibody, HRP	BIORad	31430
goat-anti-rabbit-igg-h-l-hrp-conjugate	BIORad	1706515
AF488, secondary antibody	Dianova	115-545-146
Cy5, secondary antibody	Dianova	111-175-144
Alpha-Tubulin, Rabbit	Cell signaling Technology	2144S
**Cell lines**		
Vero-76	ATCC	CRL 1587
Calu-3	ATCC	HTB-55
**Chemicals**		
RPMI 1640	Lonza	BE12-115F
EMEM	Sigma Aldrich	M7278
M199	Lonza	BE12-117F
Fetal serum	Sigma Aldrich	F7524
Human serum	Sigma Aldrich	H4522
10x MEM for plaque assay medium	Gibco	21430
Endothelial growth supplement (ECGS)	Sigma	02-102
Hoechst 33342	Merck	14533
Lymphocyte separation medium	Sigma Aldrich	Histopaque® 10771
GM-CSF	Peprotech	300-03
Penicillin/streptomycin	Biozym	882082
Fluoresceinisothiocyanat-Dextran	Sigma	FD4-250MG
DMEM without phenol red	Merck	D1145-500ML
**Commercial Kits**		
CyQUANT LDH cytotoxicity assay kit	Thermo Fisher Scientific	C20301
RNeasy Mini Kit	Qiagen	74106
QIAmp viral RNA Mini Kit	Qiagen	52906
RIDA Gene SARS-CoV-2 real-time PCR kit	R-Biopharm	PG6815
Legendplex Human anti-virus response panel kit	Biolegend	740349
**Oligonucleotides**		
human_IFN-alpha_fw GACTCCATCTTGGCTGTGA	metabion	N/A
human_IFN-alpha_rev TGATTTCTGCTCTGACAACCT	metabion	N/A
human_IFNβ_fw TCTGGCACAACAGGTAGTAGGC	metabion	N/A
human_IFNβ_rev GAGAAGCACAACAGGAGAGCAA	metabion	N/A
IFN-L1_fw	metabion	N/A

CGCCTTGGAAGAGTCACTCA		
IFN-L1_rev GAAGCCTCAGGTCCCAATTC	metabion	N/A
IFN-L2/3_fw AGTTCCGGGCCTGTATCCAG	metabion	N/A
IFN-L2/3_rev GAGCCGGTACAGCCAATGGT	metabion	N/A
human_IL-6_fw CAGCCCTGAGAAAGGAGACATG	metabion	N/A
human_IL-6_rev GCATCCATCTTTTTCAGCCATC	metabion	N/A
human_IL-8_fw ATGACTTCCAAGCTGGCCGTGGCT	metabion	N/A
human_IL-8_rev TCTCAGCCCTCTTCAAAAACTTCT	metabion	N/A
human_IP-10_fw CCAGAATCGAAGGCCATCAA	metabion	N/A
human_IP-10_rev TTTCCTTGCTAACTGCTTTCAG	metabion	N/A
human_TNF-alpha_fw GGAGAAGGGTGACCGACTCA	metabion	N/A
human_TNF-alpha_rev CTGCCCAGACTCGGCAA	metabion	N/A
cIAP2_fw GGGACCAACAGGTTGTTCTGGTA	metabion	N/A
cIAP2_rev CAGAGTTATGACTCGGACGTGTTGA	metabion	N/A
TRAIL_fw GTCTCTCTGTGTGGCTGTAACTTACG	metabion	N/A
TRAIL_rev AAACAAGCAATGCCACTTTTGG	metabion	N/A
RIPK1_fw GGGAAGGTGTCTCTGTGTTTC	metabion	N/A
RIPK1_rev CCTCGTTGTGCTCAATGCAG	metabion	N/A
human_GAPDH_fw CTCTGCTCCTCCTGTTCGAC	metabion	N/A
human_GAPDH_rev CAATACGACCAAATCCGTTGAC	metabion	N/A
Zen, 2.6 (blue edition)	Carl Zeiss AG	N/A
MrBayes	v3.2	N/A
**Systems for cDNA Synthesis and qRT-PCR**		
Nano-Drop	Nano-Drop 1000 (PEQLAB Biotechnology GmbH)	N/A
Thermo cycler (for cDNA synthesis)	Peqstar (PEQLAB Biotechnology GmbH)	N/A

Realtime PCR cycler	Rotor Gene Q (QIAGEN)	N/A
Acquisition software for RNA concentration	NanoDrop 1000 V3.8.1 (ThermoFisher Scientific)	N/A
Acquisition software for qRT-PCR	Q-Rex V1.1.0.4 (QIAGEN)	N/A
Software used for subsequent data analysis	Microsoft Office 2010 (Microsoft) GraphPad Prism V8.3.0.538 (Graphpad Software, Inc.)	N/A
**Systems for microscopy**		N/A
Model	Carl Zeiss Observer.Z1	N/A
Type of objective lenses	Plan-Apochromat	N/A
Magnification of objective lenses	20x	N/A
Numercial aperture of objective lenses	0.8	N/A
Imaging medium	Dako Fluorescence Mounting Medium	N/A
camera	Zeiss Axioxam 503 mono	N/A
ApoTome	ApoTome.2 Carl Zeiss	N/A
Aquisition software	Zeiss Zen 2.6 (blue edition)	N/A
Operations involved (deconvolution, Fourier-Filter, gamma)	Deconvolution, gamma correction, phase error correction	N/A
File formats	16-bit	N/A
**Sample Preparation, Sequencing and Analysis**		
MinION - Sequencer	Oxford Nanopore Technologies	N/A
Ligation Sequencing Kit (SQK-LSK109)	Oxford Nanopore Technologies	N/A
R9.4.1 flow cells Nanopore FLO-MIN106	Oxford Nanopore Technologies	N/A
Native Barcoding Expansion (EXP-NBD104)	Oxford Nanopore Technologies	N/A
Workflow: poreCov via nextflow using docker	For genome reconstruction (version 0.2)	https://github.com/replikation/poreCov

Primer Scheme V3 was used for amplicon-based sequencing of SARS-CoV-2	V3	https://github.com/artic-network/artic-ncov2019/blob/master/primer_schemes/nCoV-2019/V3/nCoV-2019.tsv
NEBNext Ultra II End-prep	NEB	M0493S
NEBNext Quick Ligation Module	NEB	E6056S
Q5 Hot Start HF Polymerase	NEB	M0493S
RNase OUT (125 rxn)	Thermofischer	10777019
Random Hexamers (50 μM)	Thermofischer	N8080127
Zymo Quick-RNA Viral Kit	Zymo	R1034
SuperScript IV (50 rxn)	Thermofischer	18090050
dNTP mix (10 mM each)	Thermofischer	R0192
