# Supplementary material for: SARS-CoV-2 Causes Severe Epithelial Inflammation and Barrier Dysfunction
Source: J Virol. 2021 Apr 26;95(10):e00110-21. doi: 10.1128/JVI.00110-21 (PMC8139673; doi:10.1128/JVI.00110-21)
Supplement: Supplemental file 2 [file JVI.00110-21-s0002.pdf]

Figure S1

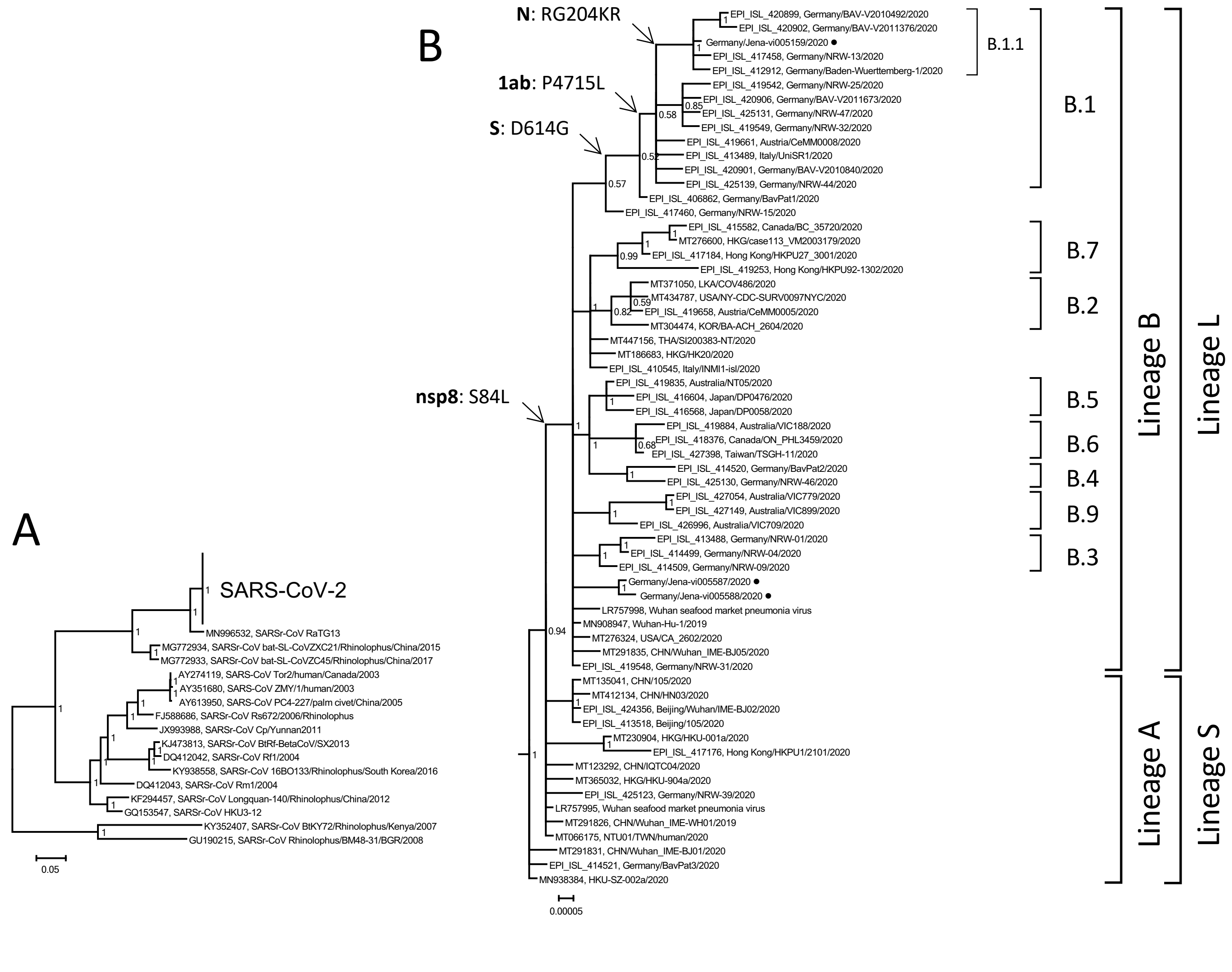

B
N: RG204KR
1ab: P4715L
S: D614G
nsp8: S84L
EPI_ISL_420899, Germany/BAV-V2010492/2020
EPI_ISL_420902, Germany/BAV-V2011376/2020
Germany/Jena-vi005159/2020 ●
EPI_ISL_417458, Germany/NRW-13/2020
EPI_ISL_412912, Germany/Baden-Wuerttemberg-1/2020
EPI_ISL_419542, Germany/NRW-25/2020
EPI_ISL_420906, Germany/BAV-V2011673/2020
EPI_ISL_425131, Germany/NRW-47/2020
EPI_ISL_419549, Germany/NRW-32/2020
EPI_ISL_419661, Austria/CeMM0008/2020
EPI_ISL_413489, Italy/UniSR1/2020
EPI_ISL_420901, Germany/BAV-V2010840/2020
EPI_ISL_425139, Germany/NRW-44/2020
EPI_ISL_406862, Germany/BavPat1/2020
EPI_ISL_417460, Germany/NRW-15/2020
EPI_ISL_415582, Canada/BC_35720/2020
MT276600, HKG/case113_VM2003179/2020
EPI_ISL_417184, Hong Kong/HKPU27_3001/2020
EPI_ISL_419253, Hong Kong/HKPU92-1302/2020
MT371050, LKA/COV486/2020
MT434787, USA/NY-CDC-SURV0097NYC/2020
EPI_ISL_419658, Austria/CeMM0005/2020
MT304474, KOR/BA-ACH_2604/2020
MT447156, THA/SI200383-NT/2020
MT186683, HKG/HK20/2020
EPI_ISL_410545, Italy/INMI1-isl/2020
EPI_ISL_419835, Australia/NT05/2020
EPI_ISL_416604, Japan/DP0476/2020
EPI_ISL_416568, Japan/DP0058/2020
EPI_ISL_419884, Australia/VIC188/2020
EPI_ISL_418376, Canada/ON_PHL3459/2020
EPI_ISL_427398, Taiwan/TSGH-11/2020
EPI_ISL_414520, Germany/BavPat2/2020
EPI_ISL_425130, Germany/NRW-46/2020
EPI_ISL_427054, Australia/VIC779/2020
EPI_ISL_427149, Australia/VIC899/2020
EPI_ISL_426996, Australia/VIC709/2020
EPI_ISL_413488, Germany/NRW-01/2020
EPI_ISL_414499, Germany/NRW-04/2020
EPI_ISL_414509, Germany/NRW-09/2020
Germany/Jena-vi005587/2020 ●
Germany/Jena-vi005588/2020 ●
LR757998, Wuhan seafood market pneumonia virus
MN908947, Wuhan-Hu-1/2019
MT276324, USA/CA_2602/2020
MT291835, CHN/Wuhan_IME-BJ05/2020
EPI_ISL_419548, Germany/NRW-31/2020
MT135041, CHN/105/2020
MT412134, CHN/HN03/2020
EPI_ISL_424356, Beijing/Wuhan/IME-BJ02/2020
EPI_ISL_413518, Beijing/105/2020
MT230904, HKG/HKU-001a/2020
EPI_ISL_417176, Hong Kong/HKPU1/2101/2020
MT123292, CHN/IQTC04/2020
MT365032, HKG/HKU-904a/2020
EPI_ISL_425123, Germany/NRW-39/2020
LR757995, Wuhan seafood market pneumonia virus
MT291826, CHN/Wuhan_IME-WH01/2019
MT066175, NTU01/TWN/human/2020
MT291831, CHN/Wuhan_IME-BJ01/2020
EPI_ISL_414521, Germany/BavPat3/2020
MN938384, HKU-SZ-002a/2020
0.00005
B.1.1
B.1
B.7
B.2
B.5
B.6
B.4
B.9
B.3
Lineage B
Lineage L
Lineage A
Lineage S
A
SARS-CoV-2
MN996532, SARSr-CoV RaTG13
MG772934, SARSr-CoV bat-SL-CoVZXC21/Rhinolophus/China/2015
MG772933, SARSr-CoV bat-SL-CoVZC45/Rhinolophus/China/2017
AY274119, SARS-CoV Tor2/human/Canada/2003
AY351680, SARS-CoV ZMY/1/human/2003
AY613950, SARS-CoV PC4-227/palm civet/China/2005
FJ588686, SARSr-CoV Rs672/2006/Rhinolophus
JX993988, SARSr-CoV Cp/Yunnan2011
KJ473813, SARSr-CoV BtRf-BetaCoV/SX2013
DQ412042, SARSr-CoV Rf1/2004
KY938558, SARSr-CoV 16BO133/Rhinolophus/South Korea/2016
DQ412043, SARSr-CoV Rm1/2004
KF294457, SARSr-CoV Longquan-140/Rhinolophus/China/2012
GQ153547, SARSr-CoV HKU3-12
KY352407, SARSr-CoV BtKY72/Rhinolophus/Kenya/2007
GU190215, SARSr-CoV Rhinolophus/BM48-31/BGR/2008
0.05

Figure S2

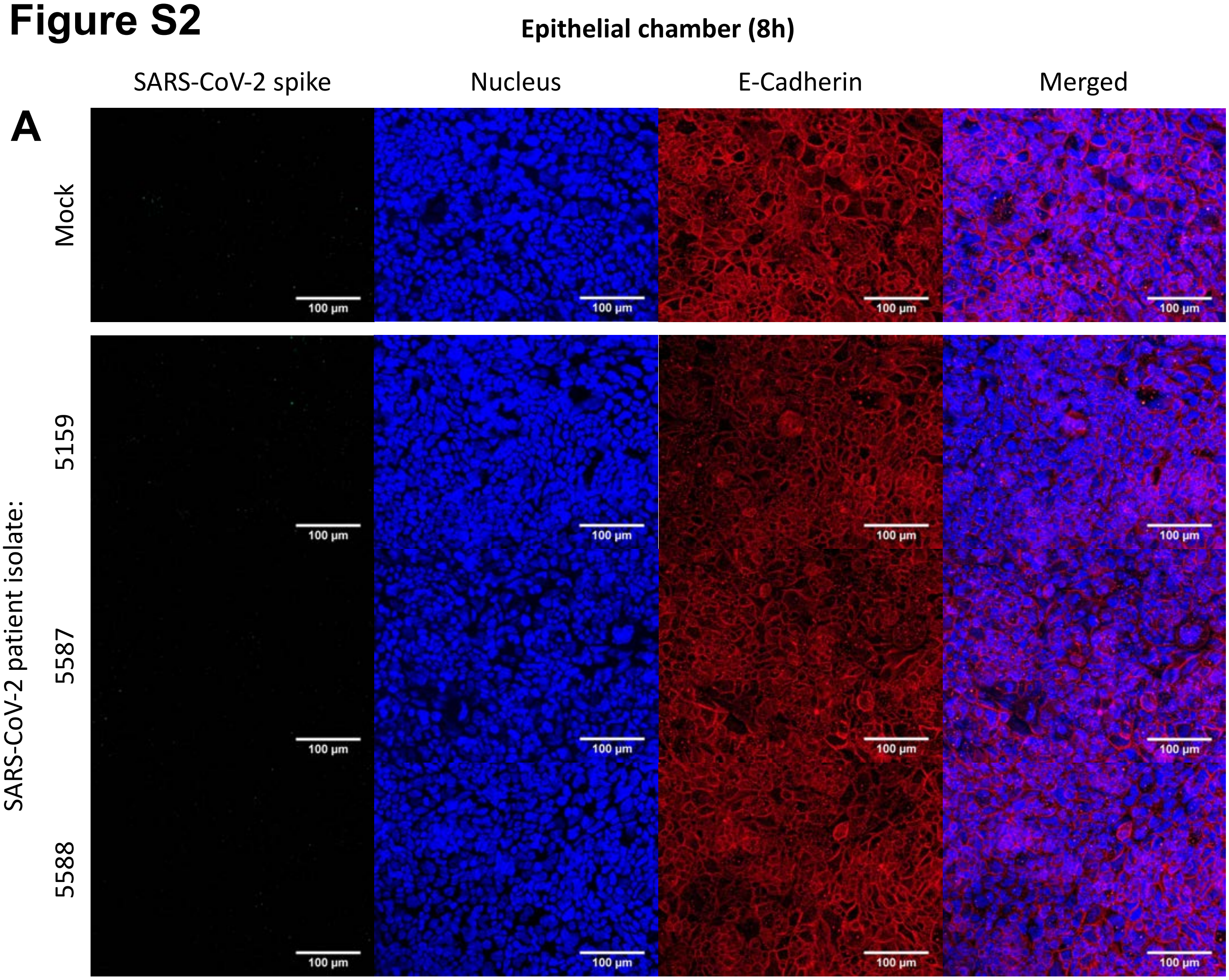
Epithelial chamber (8h)
SARS-CoV-2 spike
Nucleus
E-Cadherin
Merged
A
Mock
SARS-CoV-2 patient isolate:
5159
5587
5588
100 µm

Figure S2

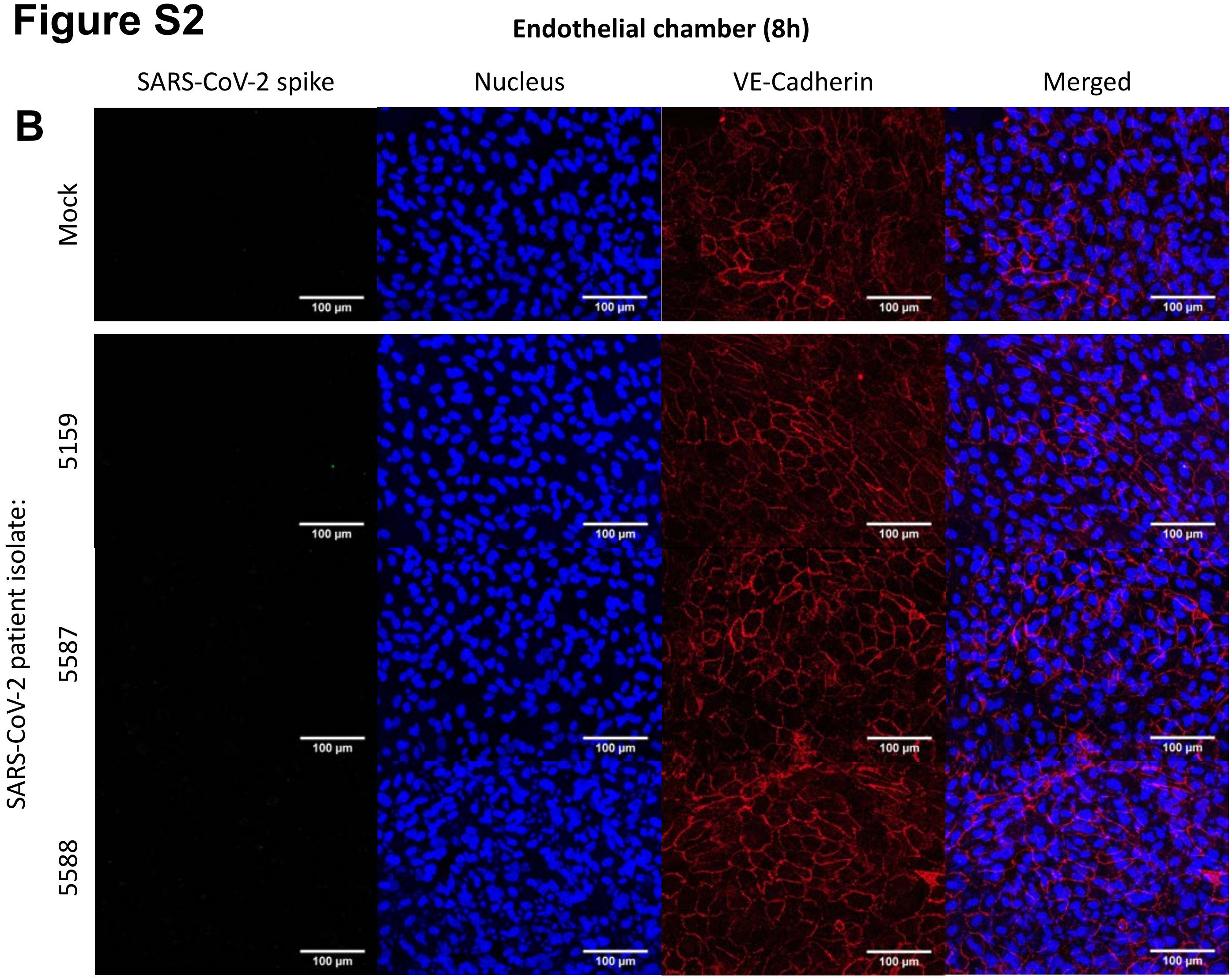
Endothelial chamber (8h)
SARS-CoV-2 spike
Nucleus
VE-Cadherin
Merged
B
Mock
5159
5587
5588
SARS-CoV-2 patient isolate:
100 µm

Figure S2

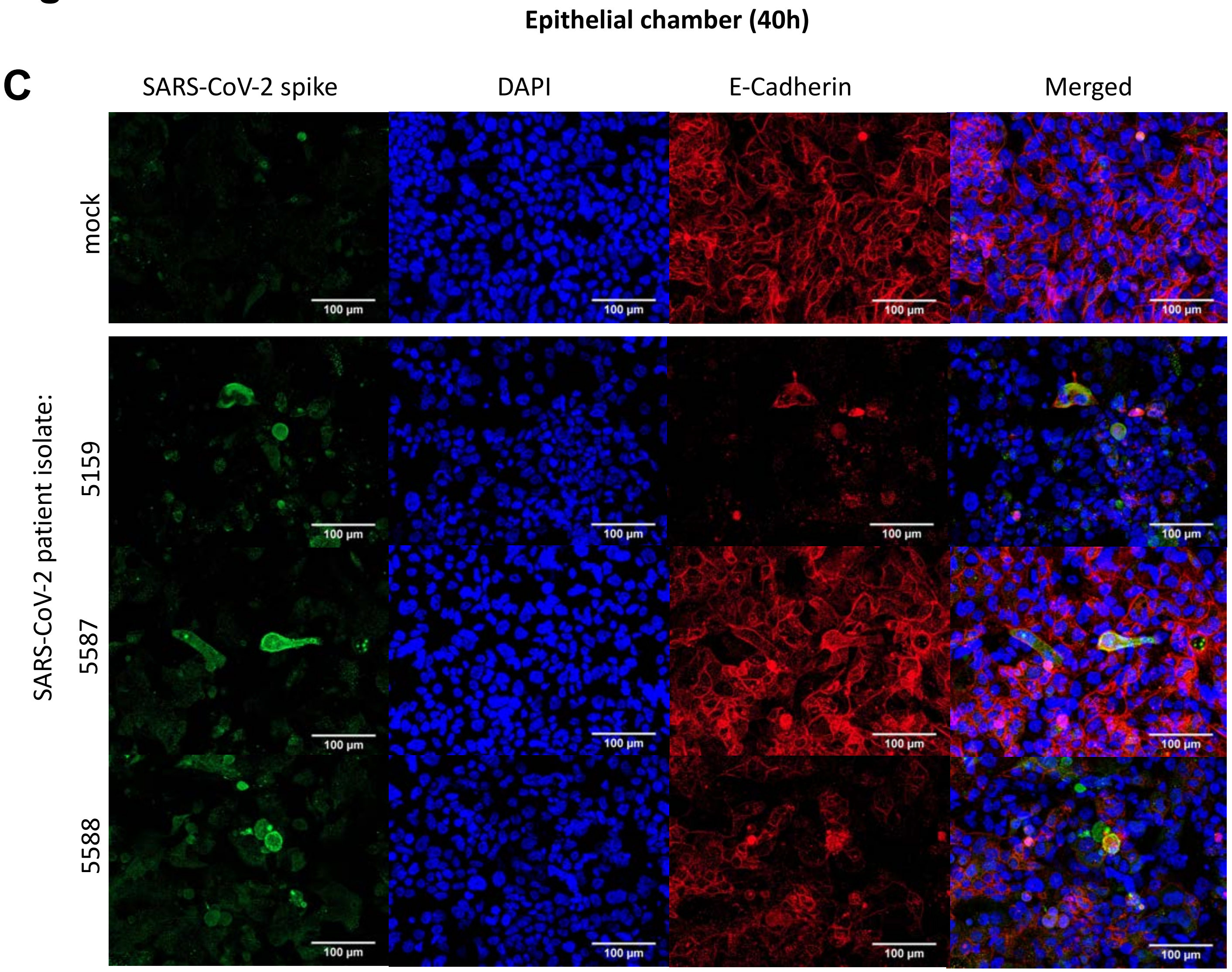
Epithelial chamber (40h)
C
SARS-CoV-2 spike
DAPI
E-Cadherin
Merged
mock
SARS-CoV-2 patient isolate:
5159
5587
5588
100 µm

Figure S2

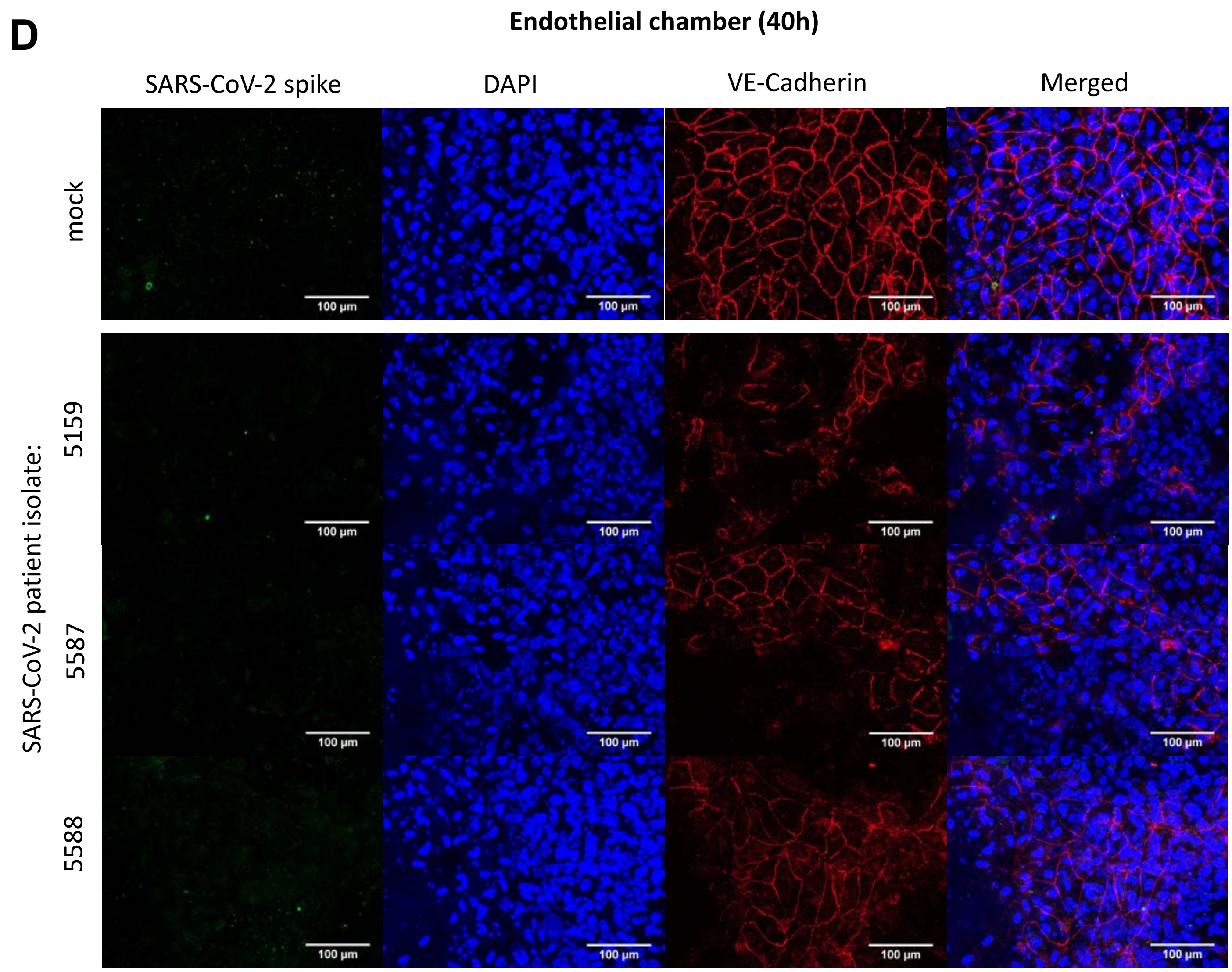
D
Endothelial chamber (40h)
SARS-CoV-2 spike
DAPI
VE-Cadherin
Merged
mock
SARS-CoV-2 patient isolate:
5159
5587
5588
100 µm

Figure S1: Phylogenetic tree of SARS-CoV-2.

(A) Phylogenetic analysis revealed a close relationship of SARS-CoV-2 to the SARS-related coronaviruses RaTG13, bat-SL-CoVZXC21 and bat-SL-CoVZC45. Sequences of strains 5587 and 5588 exhibit two base substitutions T8,782C (**nsp1ab:** synonymous) and C28,144T (**nsp8:** S84L). (B) Accordingly, 5587 and 5588 clustered with lineage L/lineage B strains in the phylogenetic analysis. Both strains exhibit deletion of **nsp1ab** D448 and two synonymous substitutions (T514C, C5512T). Beside the **nsp8** S84L substitution, strain 5159 has accumulated three additional amino acid substitutions (**S:** D614G, **nsp1ab:** P4715L and **N:** R203K/G204R) which place this virus in lineage B.1.1.

Figure S2: Infection with SARS-CoV-2 results in the disruption of the epithelial- and endothelial barrier.

The epithelial side of the chip model was left uninfected (mock) or infected with three different SARS-CoV-2 patient isolates (5159, 5587, 5588) (MOI=1). Immunofluorescence staining was performed 8h (A, B) and 40h p.i., (C, D). The E-cadherin of the epithelial layer (A, C) and the VE-cadherin of the endothelial layer (B, D) were visualized by an anti-E-Cadherin-specific antibody or an anti-VE-Cadherin antiserum, respectively, and a Cy5 goat anti-rabbit IgG (red). (A-D) The SARS-CoV-2 was visualized by detection of the spike protein via a spike-specific antibody and an Alexa Fluor™ 488-conjugated goat anti-mouse IgG (green). The nuclei were stained with Hoechst 33342 (blue). Scale bars represent 100 µm.
